# Transcriptomic Profiling and Physiological Analysis of *Haloxylon ammodendron* in Response to Osmotic Stress

**DOI:** 10.3390/ijms19010084

**Published:** 2017-12-29

**Authors:** Hui-Juan Gao, Xin-Pei Lü, Ling Zhang, Yan Qiao, Qi Zhao, Yong-Ping Wang, Meng-Fei Li, Jin-Lin Zhang

**Affiliations:** 1 State Key Laboratory of Grassland Agro-Ecosystems, College of Pastoral Agriculture Science and Technology, Lanzhou University, Lanzhou 730020, China; gaohj15@lzu.edu.cn (H.-J.G.); lvxp17@lzu.edu.cn (X.-P.L.); lzhang12@lzu.edu.cn (L.Z.); zhaoq@lzu.edu.cn (Q.Z.); wangyp16@lzu.edu.cn (Y.-P.W.); 2 College of Agriculture and Forestry, Longdong University, Qingyang 745000, China; yanqiao@ldxy.edu.cn; 3 College of Life Science and Technology, Gansu Agricultural University, Lanzhou 730070, China; lmf@gsau.edu.cn

**Keywords:** *Haloxylon ammodendron*, osmotic stress, molecular responses, regulation pathways, physiological analysis, osmoprotectants

## Abstract

*Haloxylon ammodendron*, a perennial xero-halophyte, is an essential species for investigating the effects of drought on desert tree. To gain a comprehensive knowledge on the responses of *H. ammodendron* to drought stress, we specially performed the molecular and physiological analysis of *H. ammodendron* in response to −0.75 MPa osmotic stress for six and 24 h in lab condition via RNA-seq and digital gene expression (DGE). In total, 87,109 unigenes with a mean length of 680 bp and 13,486 potential simple sequence repeats (SSRs) were generated, and 3353 differentially expressed genes (DEGs) in shoots and 4564 in roots were identified under stress. These DEGs were mainly related to ion transporters, signal transduction, ROS-scavenging, photosynthesis, cell wall organization, membrane stabilization and hormones. Moreover, the physiological changes of inorganic ions and organic solute content, peroxidase (POD) activity and osmotic potential were in accordance with dynamic transcript profiles of the relevant genes. In this study, a detailed investigation of the pathways and candidate genes identified promote the research on the molecular mechanisms of abiotic stress tolerance in the xero-halophytic species. Our data provides valuable genetic resources for future improvement of forage and crop species for better adaptation to abiotic stresses.

## 1. Introduction

Drought is one of the major abiotic stresses affecting plant growth and development worldwide, leading to productivity reduction of approximately one third of earth’s arable land [1,2]. The regions of desertification occupy about 35% of the earth’s land, and 22% of land in China [3]. In addition, global surveys suggested that drought induced an increasing number of tree mortality [4]. Xerophytes, growing in arid regions, have various multiple protective mechanisms to cope with stress conditions in the process of long-term evolution [5,6]. Therefore, understanding the regulatory mechanism involved in stress responses and identifying stress-resistant genes in xerophytes are necessary to improve drought tolerance of crop plants using genetic engineering.

*Haloxylon ammodendron*, a C4 perennial succulent xero-halophytic shrub with excellent tolerance to drought and salinity, is naturally distributed in saline land, gravel desert and clay desert in Asia and Africa [7,8]. In China, *H. ammodendron* was found in the northwest desert land, mostly in Xinjiang, Inner Mongolia, Gansu and Qinghai provinces [9]. *H. ammodendron* plays vital roles in the ecological restoration and maintenance of the structure and function of the ecosystem via sand fixation, water conservation and constructing agriculture and pasture shelterbelts [10]. *H. ammodendron* contains abundant nutrient contents and its photosynthesized stems and fruits have high palatability; additionally, the “ginseng of the desert” (*Cistanche deserticola* Y. C. Ma), which is a completely non-photosynthetic desert-broomrape, often parasitizes the roots of *H. ammodendron*, making *H. ammodendron* an important forage and tonic shrub [11]. Up to now, the research advances on *H. ammodendron* have mainly focused on its structural and physiological changes in response to drought stress: (1) *H. ammodendron* could absorb a large number of Na^+^ than K^+^ transported to photosynthesizing stems and leaves for osmotic adjustment under drought stress [12]; (2) 1.0% of the soil water was considered to be the “survival water threshold” of the growth [8]; (3) the application of Na or the combination usage of Na and Si led to enhancement of growth and drought tolerance of *H. ammodendron* [10]. However, the physiological and molecular mechanism underlying how *H. ammodendron* adapt to dry environment is still unclear and the genome of *H. ammodendron* has not been sequenced up to now.

Since the advent and development of next-generation sequencing technologies, namely Illumina/Solexa and Roche/454, increasing RNA-seq methods were used to identify specific stress responsive genes and regulatory networks at transcriptional level [13]. DEGs (23,429) involved in transport, transcription, cell signal transduction, abscisic acid (ABA) regulation and metabolism were discovered in *Populus euphratica* in response to salt stress [14]. DEGs associated with organic cation transporting, oxidoreductases and abiotic stress were only observed in *Populus euphratica* compared with sensitive *Populus trichocarpa* [15]. DEGs (1239) only responding to moderate dehydration and 4135 DEGs specifically responding to desiccation were identified in *Boea hygrometrica*, a resurrection model plant [16]. 18,111 down-regulated contigs mostly related to metabolic and cell part and 17,236 up-regulated contigs related to abiotic stresses and death were identified in a California oak species, *Quercus lobata* Née [17]. DEGs mainly responsible for stress response, transport and hormone signal were overlapped in *Populus simonii* under high temperature or drought stresses, and the physiological changes of carbohydrates, antioxidants and hormone were linked with the transcript profile [18].

In the present study, in order to obtain a more complete understanding of the complex gene networks regulating physiological and biochemical processes for *H. ammodendron* adaptation to osmotic stress, we specially examined the gene expression dynamics of plants responding to drought and elucidated the potential molecular mechanisms involved in drought tolerance of *H. ammodendron*. The obtained information will be facilitative to develop stress-tolerant crops using drought-tolerance-relevant genes in *H. ammodendron* through genetic manipulation.

## 2. Results

### 2.1. De Novo Transcriptome Assembly of Transcriptome

Illumina HiSeq™ 2000 platform generated a total of 69,414,094 and 69,232,936 clean reads from shoot and root, respectively (Table S1). Then, we used paired-end information to join the contigs into scaffolds and further assemblies, generating 82,736 unigenes for shoot and 99,624 for root. After further analysis, we obtained the final assembly which consisted of 87,109 gap-free unigenes with a mean length of 680 bp (Table 1). The size distribution of the unigenes showed that 20,175 unigenes were greater than 1000 bp in length (Figure S1). These results suggested that the *H. ammodendron* transcriptome was of high quality in our experiment. 

### 2.2. Functional Annotation of H. ammodendron Transcriptome

Unigenes (87,109) were then annotated by BLASTx. As a result, 50,452 unigenes obtained a functional annotation (Table S2). Moreover, 80% of the 1500–2000 bp, and 80% of the query sequences greater than 2000 bp were matched successfully with the Nr (non-redundant protein database) (Figure S2A). For E-value distribution, 44.8% genes had a threshold E-value less than 1.0 × 10^−45^ (Figure S2B). For similarity distribution, 45.9% unigenes had similarity ranged between 17–60% (Figure S2C). For species distribution, *Vitis vinifera* had the highest homology of 23.6% with *H. ammodendron* (Figure S2D). 

We used gene ontology (GO) assignments to classify the possible functions of the annotated genes. For biological process (BP), “cellular process” was highly represented; for cellular component (CC), “cell” was mainly dominant; for molecular function (MF), genes associated with “binding and catalytic activity” were highly clustered (Figure S3). In addition, phylogenetic classification was performed according to clusters of orthologous groups (COG), and 19,196 unigenes were grouped into 25 functional classes (Figure S4). We further used Kyoto encyclopedia of genes and genomes (KEGG) classification to perform biological pathways analysis, and totally 28,794 unigenes were assigned to 128 KEGG pathways. The top most represent pathway was the global and overview maps, followed by translation and carbohydrate metabolism (Figure S5).

### 2.3. SSRs in the Transcriptome in H. ammodendron

**Figure 1:**
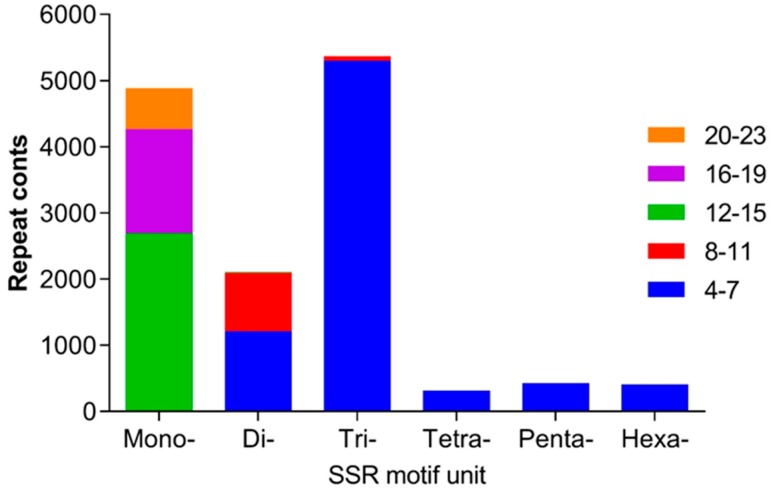
Distribution of simple sequence repeats (SSRs) in the transcriptome of *H. ammodendron*.

Simple sequence repeats (SSRs) were detected in the 87,109 unigenes of *H. ammodendron* with MISA software. In total, 13,486 SSRs with 1–6 repeat units and distributed in 1887 sequences were identified, among which the tri-nucleotide repeat was the most abundant type with a ratio of 39.7%, followed by mono-nucleotide (36.2%). In addition, the SSRs with 4–7 tandem repeats were absolutely predominant, followed by 12–15 and 16–19 tandem repeats (Figure 1).

### 2.4. Identification of DEGs in H. ammodendron under Osmotic Stress

**Figure 2:**
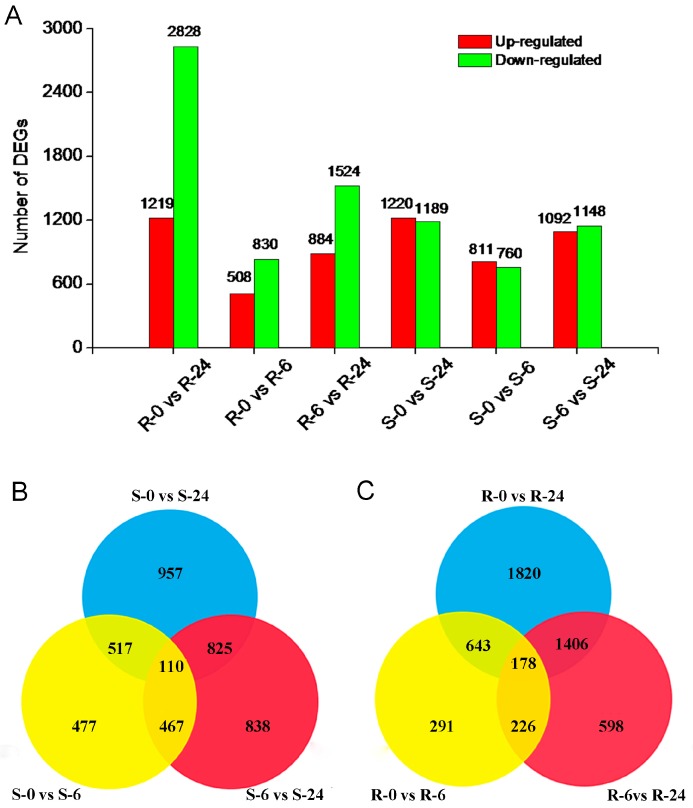
Summary of differentially expressed genes (DEGs) under 6 and 24 h sorbitol treatment; (**A**) Summary of the numbers of up- and down-regulated DEGs in shoots and roots; (**B**) DEGs expressed at random two time point in shoots; (**C**) DEGs expressed at random two time point in roots.

Six cDNA libraries were sequenced by the Illumina DGE tag method, and mapped to the transcriptome reference database (Table S3). In shoots, compared with the control, 3353 DEGs were observed totally, and there were 1571 and 2409 DEGs in S-6 and S-24, respectively. Among these genes, 627 DEGs were expressed throughout treatments (Figure 2A,B). For roots, compared with the control, 4564 DEGs were observed totally, and there were 1338 and 4047 DEGs in R-6 and R-24, respectively; among these genes, 821 DEGs were expressed throughout treatment (Figure 2A,C).

**Figure 3:**
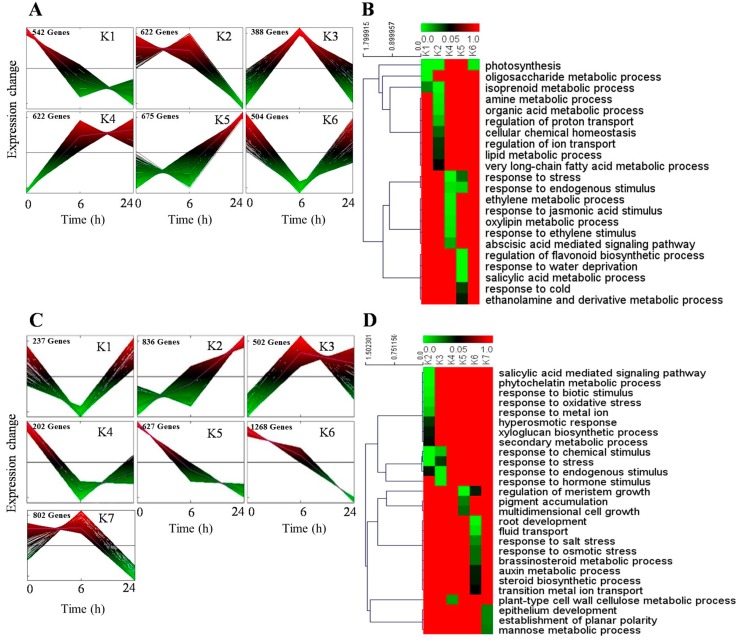
Expression profiles and enriched metabolic or signal transduction pathways of DEGs. (**A**) Expression profiles of DEGs in shoots under six and 24 h sorbitol treatment. The transcripts per million (TPM) values were transformed into z-scores, the red indicate higher expression levels and the green indicate lower expression levels; (**B**) enriched metabolic or signal transduction pathways of DEGs in shoots. The green and red color indicate the *p* value of significantly enriched pathways, green (*p* = 0) indicate the dominantly enriched pathway; (**C**) expression profiles of DEGs in roots under 6 and 24 h sorbitol treatment. The TPM values were transformed into z-scores, the red indicate higher expression levels and green indicate lower expression levels; (**D**) enriched metabolic or signal transduction pathways of DEGs in roots. The green and red color indicate the *p* value of significantly enriched pathways, green (*p* = 0) indicate the dominantly enriched pathway.

Then, K-Means/K-Medians Support Module (KMS) embedded in the MEV program was used to cluster the total of 3353 and 4564 DEGs in shoots and roots into six and seven profiles (*p* < 0.05), respectively (Figure 3A,C). Pearsons correlation co-efficient was used to identify significantly enriched metabolic or signal transduction pathways of DEGs by recording the coefficient of variation (CV) of each DEGs. For shoots, K1 was mainly involved in photosynthesis and oligosaccharide metabolic process; K2 was related to photosynthesis and isoprenoid metabolic process; K4 was associated with stress, ethylene and jasmonic acid stimulus response; K5 participates in water deprivation, salicylic acid metabolic and flavonoid biosynthesis; K6 was associated with photosynthesis (Figure 3B). For roots, K2 was mainly involved in the salicylic acid signaling pathway, phytochelatin metabolic process and stress response; K3 was related to endogenous and hormone stimulus response; K5 was associated with regulation of meristem growth; K6 was mainly involved in root development and fluid transport (Figure 3D).

**Figure 4:**
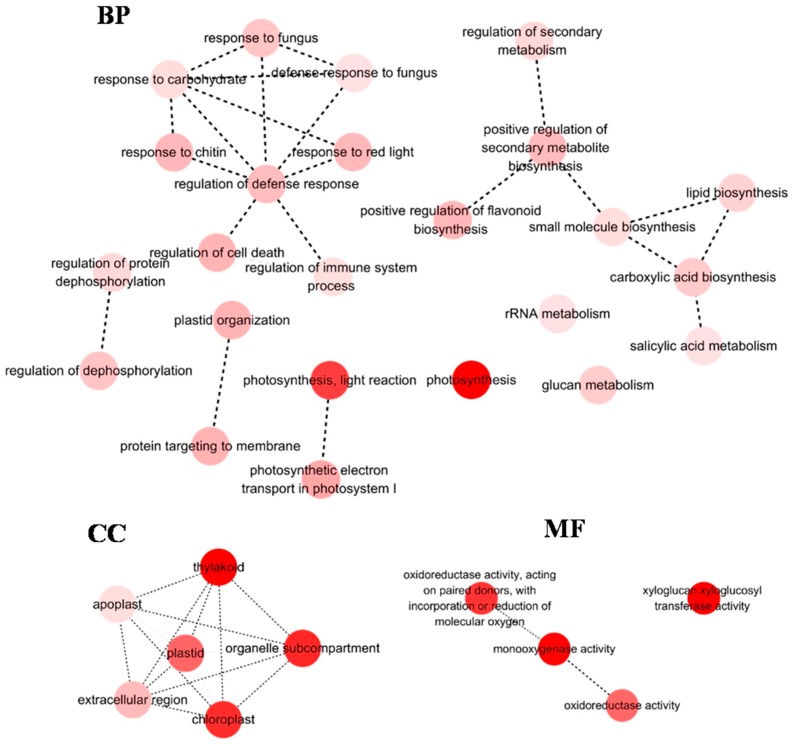
Gene ontology (GO) terms enrichment analysis of DEGs (24 h) in shoot. Few GO terms were over-represented in S6 libraries. Therefore, only GO terms enrichment in S24 libraries were displayed. The genes were assigned to three main categories: biological process (BP), molecular function (MF) and cellular component (CC). Node filled color represented *p* value. White nodes were not significantly over-represented terms.

**Figure 5:**
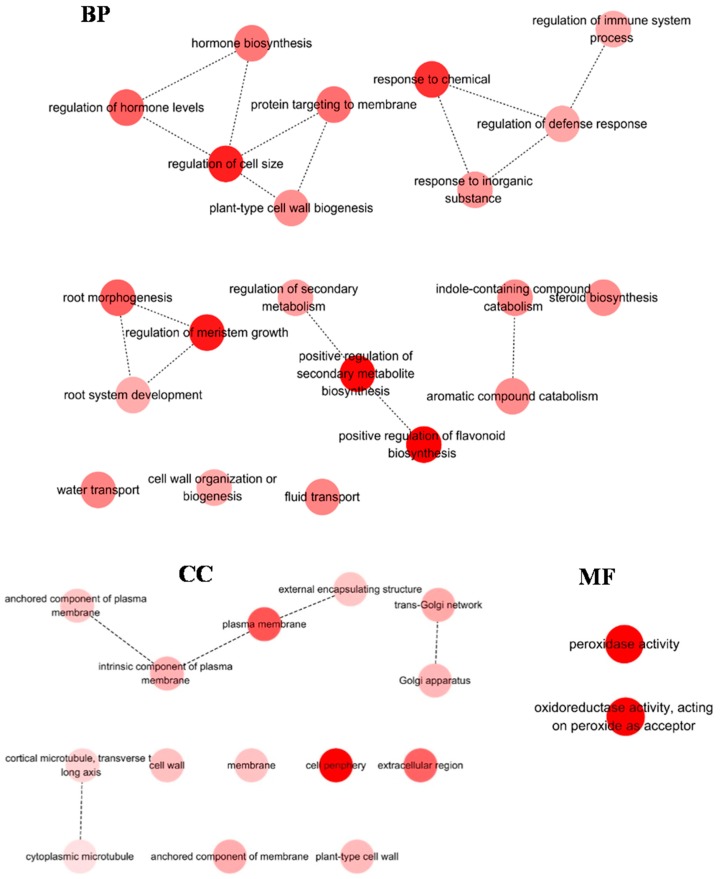
GO terms enrichment analysis of DEGs (24 h) in root. Few GO terms were over-represented in R6 libraries. Therefore, only GO terms enrichment in R24 libraries were displayed. The genes were assigned to three main categories: biological process (BP), molecular function (MF) and cellular component (CC). Node filled color represented *p* value. White nodes were not significantly over-represented terms.

Analysis for GO enrichment of DEGs was carried out using Cytoscape (*p* < 0.01). Few GO terms were over-represented in six hours. Therefore, only GO enrichment in 24 h was displayed. In shoots, “photosynthesis” was the most represented in BP, followed by “light reaction”; for CC, “thylakoid” was most dominant, followed by “chloroplast”; for MF, “monooxygenase activity” and “xyloglucan: xyloglucosyl transferase activity” were dominantly enriched (Figure 4). In roots, “positive regulation of secondary metabolite biosynthesis” was mainly clustered in BP; for CC, “cell periphery” was most represent, followed by “plasma membrane”; for MF, “peroxidase activity” and “oxidoreductase activity” were dominantly enriched (Figure 5).

### 2.5. Screening and Characterization of the DEGs Related to Adaptation to Osmotic Stress in H. ammodendron

#### 2.5.1. DEGs Related to Transporters

In shoots, genes related to ion transport (*Arabidopsis* K^+^ transporter 1 (AKT1), Mkt1p, cyclic nucleotide-gated channel-17 (CNGC-17), ER-type calcium ATPase, calcium antiporter 1 and vacuolar H^+^-pyrophosphatase) were highly induced under osmotic stress with four genes up-regulated. The down-regulation of three genes (vacuolar iron transporter, nitrate transporter 1.1 and cation/H^+^ antiporter 20-like) were observed in roots (Table S4).

#### 2.5.2. DEGs Related to Signal Transduction 

In shoots, plenty of genes homologous to transcription factors (TF) were differential expressed under osmotic stress, with 18 genes up-regulated and 16 down-regulated. In roots, nine genes were up-regulated and one down-regulated. Various kinase and phosphatase genes were also found differentially expressed. The up-regulation of 24 genes and down-regulation of eight genes in shoots and the up-regulation of five genes and down-regulation of two genes in roots were observed (Table S5). Hormone pathways play crucial roles in abiotic stresses during plant growth and development. In shoots, the abundance of five genes involved in ABA and ethylene synthesis, two genes associated with phytosulfokine, six genes involved in jasmonic acid (JA) biosynthesis and one gene involved in cytokinin were increased. In roots, four genes were down-regulated (Table S5).

#### 2.5.3. DEGs Related to Reactive Oxygen Species (ROS)

In shoots, the up-regulation of 11 genes related to ROS-scavenging were induced, while a gene encoding peroxidase precursor was down-regulated. In roots, five genes were up-regulated (Table S6).

#### 2.5.4. DEGs Related to General Metabolism

In this study, many DGEs were classified into photosynthetic responsive genes, including ten up-regulated genes and 16 down-regulated genes in shoots (Table S7). Eighteen genes related to cell wall organization were more abundant under osmotic stress in shoots, while pectinesterase inhibitor was down-regulated. In addition, five up-regulated genes involved in cell membrane stability were identified. In roots, four genes involved in cell wall organization were differentially expressed (Table S7). Sixteen genes classified into secondary metabolism were differentially expressed in shoots under osmotic stress, two genes involved in pigment synthesis and six genes for cytochrome P450 were up-regulated. In roots, one gene for 2-nitropropane dioxygenase family and a gene for cytochrome P450 were up-regulated, and one gene for geranylgeranyl diphosphate reductase was down-regulated (Table S7).

#### 2.5.5. General Stress Response Genes

The expression of dehydration-responsive genes was significantly altered. In shoots, sixteen genes were highly up-regulated. While three genes were down-regulated. In roots, four genes were up-regulated and seven genes were down-regulated. Additionally, a plethora of genes were classified into pathogenesis-related protein. Fifteen of the 22 genes were ranked as positive DEGs in shoots. In roots, there were 5 pathogenesis-related genes differently expressed with three up-regulated (Table S8).

### 2.6. Validation of the DEGs through qRT-PCR

**Figure 6:**
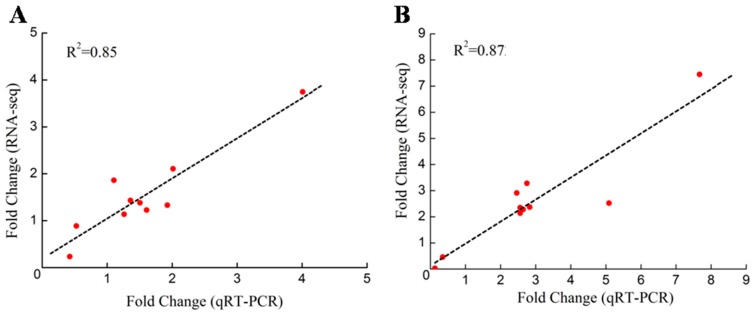
Confirmation of the expression profiles of ten randomly selected unigenes by qRT-PCR. (**A**) A linear regression analysis of the fold-change (6 h vs. 0 h) between the DGE and the qRT-PCR; (**B**) a linear regression analysis of the fold-change (24 h vs. 0 h) between the DGE and the qRT-PCR. Total RNA was extracted from three replicated samples individually for each treatment of shoots and roots (*n* = 3).

To further test the reliability of the transcriptome analysis results, ten DEGs were randomly selected for quantitative reverse transcription polymerase chain reaction (qRT-PCR) analysis (Figure S6). The fold changes of relative gene expression by qRT-PCR were consistent with their DGE profiling. A linear regression analysis of the fold-changes between DGE profiling and qRT-PCR exhibited a significantly positive correlation with *R*^2^ = 0.8516 for 6 h vs. 0 h (Figure 6A) and *R*^2^ = 0.8724 for 24 h vs. 0 h (Figure 6B), indicating a very strong accuracy of our RNA-seq results.

### 2.7. Changes of Physiological Parameters under Osmotic Stress

**Figure 7:**
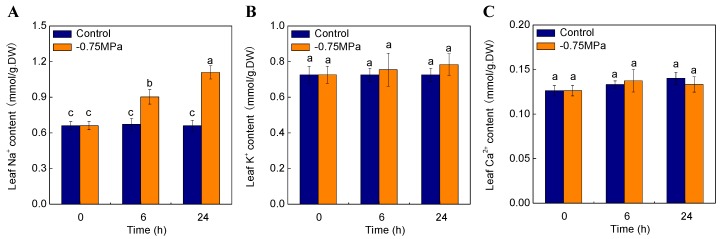
Effects of sorbitol treatment on Na^+^ (**A**), K^+^ (**B**) and Ca^2+^ (**C**) content. Values are means and bars indicate SDs (*n* = 6). Columns with different letters indicate significant difference by Duncan’s multiple range tests at *p* < 0.05.

With the prolongation of osmotic treatment (−0.75 MPa), shoot Na^+^ content increased dramatically. After 6 h and 24 h of treatment, Na^+^ content increased by 34% and 68% compared with control, respectively (*p* < 0.05) (Figure 7A). K^+^ content in leaf showed no significant change under osmotic stress (Figure 7B). Ca^2+^ content increased and then decreased, but not significantly (Figure 7C).

**Figure 8:**
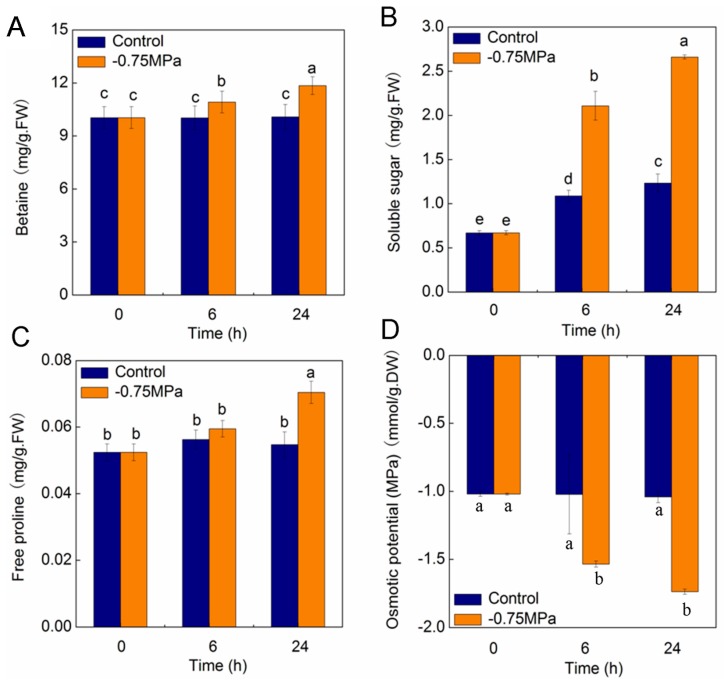
Effects of sorbitol treatment on physiological indexes: (**A**) betaine content, (**B**) soluble sugar content, (**C**) free proline content, (**D**) osmotic potential. Values are means and bars indicate SDs (*n* = 6). Columns with different letters indicate significant difference by Duncan’s multiple range tests at *p* < 0.05.

Osmotic stress (−0.75 MPa) led to a significant increase in betaine levels. After 6 h and 24 h of treatment, betaine content increased by 9% and 17% compared with control, respectively (*p* < 0.05) (Figure 8A). Osmotic stress significantly improved soluble sugar levels in shoots. After 6 h and 24 h of treatment, soluble sugar content increased by 93% and 1.16 times compared with control, respectively (*p* < 0.05) (Figure 8B). Additionally, after 24 h of treatment, proline content increased significantly by 30% compared with control (*p* < 0.05) (Figure 8C). Shoot osmotic potential decreased significantly under osmotic stress. After 6 h of treatment, osmotic potential decreased by 50% compared with control. After 24 h of treatment, osmotic potential decreased by 67% compared with control (*p* < 0.05) (Figure 8D).

**Figure 9:**
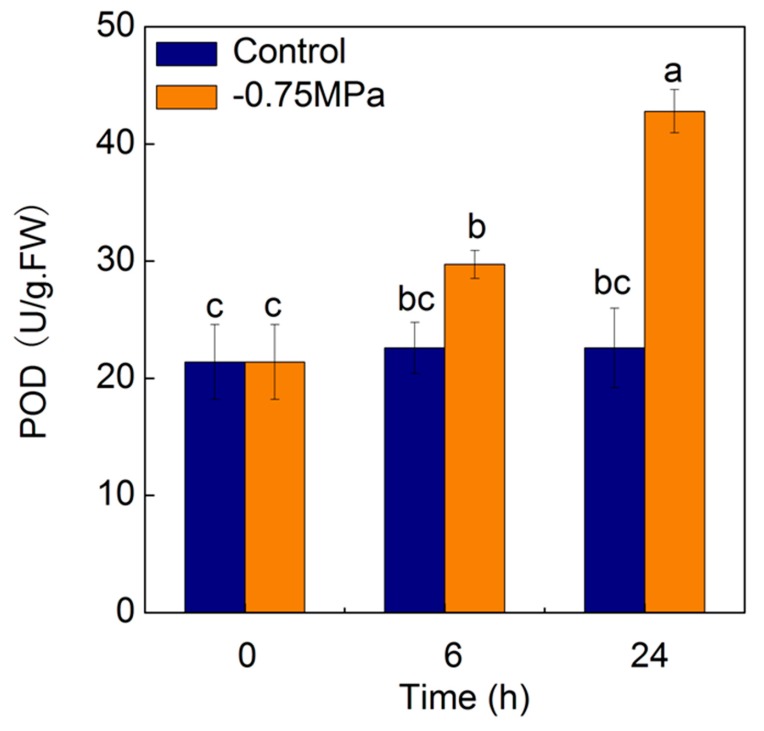
Effects of sorbitol treatment on peroxidase (POD) activity. Values are means and bars indicate SDs (*n* = 6). Columns with different letters indicate significant difference by Duncan’s multiple range tests at *p* < 0.05.

POD activity significantly increased under osmotic stress. After 6 h of treatment, POD activity increased by 31% compared with control. After 24 h of treatment, POD activity increased by 89% compared with control (*p* < 0.05) (Figure 9).

## 3. Discussion

### 3.1. Analysis of Differentially Expressed Genes

In this work, 87,109 unigenes with a mean length of 680 bp were generated and confirmed reliable by qRT-PCR, and 3353 DEGs in shoots and 4564 in roots were successfully identified in *H. ammodendron* under osmotic stress. Among the above DEGs, those expressed continuously up-regulated or down-regulated through osmotic stress were of the most interest. Many of these candidate drought-tolerance genes were involved in ion transporters, signal transduction, ROS scavenging, photosynthesis, cell wall organization, membrane stabilization, secondary metabolism and hormones, which might contribute to the adaptation of *H. ammodendron* to arid environments.

### 3.2. Up-Regulation of the Genes Related to Transporters, Signal Transduction, ROS-Scavenging, Cell Wall and Membrane Stability, Secondary Metabolism Contributed to the Adaption Ability of H. ammodendron to Osmotic Stress

Accumulating a great quantity of Na^+^ and maintaining the stability of the Na^+^/K^+^ are necessary to homeostasis of the cellular environment in *Zygophyllum xanthonylon* [19]. Mutants of *AtCNGC3* (cyclic nucleotide-gated channel) and *AtCNGC10* led to lower Na^+^ and K^+^ accumulation compared with WT under salt stress [20]. In addition, *CNGCs* could improve absorption fluxes of Ca^2+^ into plant cells [19]. The *AKT1* type potassium channel regulates uptake of Na^+^ into cell under higher salt concentrations (150 mM NaCl) [21]. The mutant of *Physcomitrella patens* with Ca^2+^-ATPase loss-of-function showed a sustained increase of Ca^2+^, while WT displayed a transient cytoplasmic Ca^2+^ signature upon salt stress [22]. Calcium antiporter 1 has been found to be involved in compartmentalization of Ca^2+^ into vacuoles [23]. Vacuolar-H^+^-PPases play a vital role in sequestering ions into vacuoles via generating the driving force [24]. In the present study, the up-regulation of *CNGCs* and *AKT1* may result in a significant accumulation of Na^+^ and slight increase of K^+^ in shoots of *H. ammodendron* under drought stress. Our results also showed the up-regulation of Ca^2+^-ATPase and down-regulation of calcium antiporter and V-H^+^-PPases, indicating that the *H. ammodendron* shoot could possess an efficient mechanism for maintaining Ca^2+^ homeostasis under drought conditions.

TFs play vital roles in regulating networks of drought-responses [25,26]. Previous studies suggested that Hsf, WRKY, NAC, MYB, GRAS, AP2 BHLH and bZIP were conducive to plants’ responses to drought and other abiotic stresses [25,26]. Moreover, over-expression of zinc finger protein gene *AtSAP5* could regulate expression of other stress-responsive genes and enhance drought tolerance of transgenic *Arabidopsis* [27]. Plant protein kinases and calmodulin-binding protein were reported to function in cellular stress signaling transduction [26,28]. CDPK, RLKs (receptor-like protein kinase) and MAPK characterized as stress responsive protein were involved in regulating signal transduction of plant growth and development [2,26,29]. Protein phosphatase 2c mediates a large amount of ABA responses [30]. In this study, the majority of genes encoding TFs, kinases and phosphatase were up-regulated in both shoots and roots, indicating an important role in the network for *H. ammodendron* to adapt to osmotic stress.

The ABA pathway was suggested to play pivotal roles in plant drought tolerance [14]; 9-*cis*-epoxycarotenoid dioxygenase, a rate-limiting enzyme in ABA biosynthesis, was identified to enhance drought and salt tolerance by increasing endogenous ABA levels [14,15,31]. DRE binding factor was found to function in ABA-independent pathways [31]. Tobacco plants transformed with zeatin *O*-glucosyltransferase gene had higher levels of the total cytokinin [32]; 1-aminocyclopropane-1-carboxylate synthase and oxidase were essential for the biosynthesis of ethylene, a vital regulator for cell elongation, senescence and defense in plants [33,34]. In addition, ethylene coordinated with NO in inducing the expression of PM H^+^-ATPase and reduction of Na^+^/K^+^ ratio for *Arabidopsis* salt tolerance [35]. Topless-related protein, 12-oxophytodienoate reductase, allene oxide synthase, allene oxide cyclase, lipoxygenase and MYC2 were demonstrated to be involved in JA biosynthesis and singling [33,36,37]. Exogenous JA could enhance *Agropyron cristatum* tolerance to osmotic stress via modulating ascorbate and glutathione metabolism [38]. It was suggested that the silencing of cytokinin dehydrogenase irreversibly degraded cytokinins into adenine/adenosine moiety, generating a higher plant yield in barley [39], and the increase of cytokinin in the transgenic rice enhanced its tolerance to osmotic stress through cytokinin-dependent sustained and coordinated interaction of carbon and nitrogen metabolism [40]. In shoots, genes related to hormones were altered, leading to higher levels of ABA, ethylene, jasmonic acid and cytokinin, and suggesting that the resistance of *H. ammodendron* to the osmotic stress is mediated by these signaling pathways.

The excessive production of ROS led to destruction of membrane systems, causing oxidative damage; however, plants have anti-oxidation mechanisms, including antioxidant enzymes, to remove ROS [19]. Antioxidant enzyme systems dominantly include glutathione metabolism, the catalase pathway, superoxide dismutase and the peroxiredoxin/thioredoxin pathway [19]. Glutathione *S*-transferase metabolism, like ascorbate peroxidase, was shown to respond to dehydration stress along with POD. Thioredoxin could scavenge H_2_O_2_ and regulate the response to oxidative stress through protein–protein interactions [41]. Alternative oxidase has the capacity to scavenge ROS under abiotic stress [42]. The specific activity of polyphenol oxidase involved in antioxidant protection increased in olive tree under cold stress [43]. In this study, the abundance of most of the genes for antioxidant enzymes was increased in shoots and root, indicating that *H. ammodendron* has strong antioxidative ability to mitigate the damages induced by ROS when subjected to osmotic stress.

The plant cell wall was believed to function as a physical and defense barrier against abiotic and biotic stresses [44]. The plasma membrane is considered as a primary site of injury from stress in plants, therefore, maintaining stabilization of the plasma membranes will aid plants to adapt to stressful environments [26]. The gene for expansin controlling cell wall extensibility was more abundant in drought-tolerant maize cultivar [45,46]. Xyloglucan endotransglucosylase/hydrolases play a crucial role in controlling cell wall extensibility and the adaptation of *Arabidopsis* to cold temperature [43,45]. Polygalacturonases was associated with degradation of pectin portion [47], and Caffeic acid 3-*O*-methyltransferase plays a pivotal role in biosynthesis of lignin [47]. Moreover, the transgenic cotton overexpressing laccase related to lignin synthesis could function as a detoxifier and degrades organic pollutants [48]. Cinnamate 4-hydroxylase (C4H) was also significantly induced under various stresses and down-regulation of C4H led to reduction of lignin accumulation in *Populus* [49]. Overexpression of myo-inositol oxygenase associated with polysaccharides significantly enhanced growth and survival rates of transgenic rice upon osmotic stress [50]. Also, lipid transfer proteins and lipase are possibly associated with the repair of stress-induced damage in membranes [51]. In the current study, the abundance of genes associated with cell wall organization and membrane stabilization were up-regulated in shoots, suggesting that maintaining the integrity of the cell wall and plasmalemma is one of essential mechanisms for *H. ammodendron* adapt to osmotic stress. In roots, C4H and lipid binding proteins were up-regulated.

It was known that chalcone synthase participating in the salicylic acid defense signaling cascades could improve the accumulation of flavonoid and isoflavonoid phytoalexins [52] and play crucial roles in safflower responding to salinity stresses [53]. Leucoanthocyanidin dioxygenase was involved in biosynthesis of proanthocyanidin and vacuole development [54]. The overexpression of homogentisate phytyltransferase, considered as a key enzyme limiting tocopherol biosynthesis in lettuce, improved the content of tocopherol, a lipid soluble compound functioning as antioxidants and involved in signal transduction and transcription regulation [55]. Cytochrome P450 (CYP450) characterized as heme-thiolate enzymes was necessary for carbon assimilation, synthesis of hormones and as a structural components of cells [56]. In addition, CYP450 monooxygenase was essential for the synthesis of lignin, pigments, defense compounds, hormones and signaling molecules [57]. In the present study, the genes related to biosynthesis of pigment and tocopherol and those encoding CYP450 were up-regulated in *H. ammodendron* responding to osmotic stress.

Threonine aldolase and Phosphoethanolamine *N*-methyltransferase were key enzymes in the biosynthesis of glycine betaine [58]. Late embryogenesis abundant protein (LEA) was associated with cellular dehydration tolerance [59], and the ectopical expression of *BhLEA1* and *BhLEA2* from *Boea hygrometrica* in transgenic tobacco could protect plant cells and stabilize photosynthetic proteins under osmotic stress [60]. Dehydrins characterized as LEAII protein also accumulated under several abiotic stresses [61]. Additionally, transgenic tobacco expressing ∆^1^-pyrroline-5-carboxylate synthetase gene resulted in a higher accumulation of proline upon a variety of environmental stresses [62]. Proteins of molecular chaperones are responsible for numerous cellular functions, such as refolding of damaged proteins and assistance of protein trafficking and degradation [63]. The transgenic rice seedlings overexpressing a small heat-shock protein 17.7 (sHSP17.7) had higher survival rate under osmotic stress and could regrow after re-watering [64]. The transgenic tobacco overexpressing chitinases generated a higher tolerance to biotic and abiotic stresses [65]. Pathogenesis-related protein, nbs-lrr resistance protein, 1,4-glucan-protein synthase, germin-like proteins, thaumatin-like protein and harpin-induced protein have been found to be involved in plant defense mechanisms [33,59,65,66,67]. In the present study, the up-regulation of genes related to betaine and proline synthesis and genes encoding LEA were identified, indicating those stress responsive genes in shoots play crucial roles in the adaptation of *H. ammodendron* to osmotic stress. In roots, the genes for toxin extrusion protein, cold acclimation protein, dehydration responsive protein, chaperone protein ClpB3, harpin-induced protein and chitinase 3 were up-regulated, suggesting that these stress responsive genes in roots play important roles for *H. ammodendron* adapt to osmotic stress.

### 3.3. Altered Expression of Genes Related to Photosynthesis Also Contributed to the Adaption of H. ammodendron to Osmotic Stress

Photosynthesis is one of the primary processes damaged during osmotic stress as a result of the alterations of photosynthetic metabolism, CO_2_ availability and subsequently oxidative stress [68]. In our study, many genes related to the Calvin cycle, photorespiration and chlorophyll biosynthesis were down-regulated, indicating that photosynthesis was inhibited in *H. ammodendron*. Additionally, overexpression of *PHD1* encoding UDP-glucose-epimerase enhanced photosynthetic rate and biomass in rice [69]. The transgenic *Anabaena* sp. line had improved photosynthetic yield with the increased activity of fructose-1,6-bisphosphate aldolase (FBA) and triosephosphate isomerase [70]. Moreover, the expression levels of photosynthesis-associated nuclear genes was improved in the *gun6-1D* mutant overexpressing plastid ferrochelatase 1 when chloroplast development was blocked in *Arabidopsis* [71]. Invertase could degrade sucrose, resulting in increased glucose and fructose [33]. The level of formate dehydrogenase catalyzing the oxidation of formate into CO_2_ and NADH was increased under various abiotic stresses [72]. The transcript of 6-phosphogluconate dehydrogenase functioning to regulate the efficiency of the pentose phosphate pathway was increased in rice during drought treatments [73]. In this study, the abundance of UDP-glucose 4-epimerase, FBA, ferrochelatase, plastid-targeted protein, photosystem II protein CP43, photoperiod responsive proteins, neutral invertase, formate dehydrogenase and 6-phosphogluconate dehydrogenase were significantly enhanced, suggesting that photosynthetic capacity could be rescued to some extent in *H. ammodendron* under osmotic stress.

### 3.4. Physiological Mechanism for H. ammodendron to Adapt to Drought Stress

It has been known that Na^+^ is the primary cause of ion-specific damage in many higher plants [74]. However, some plants exhibited remarkable growth with addition of moderate Na^+^ [74]. Na^+^ accumulated in leaves of *Zygophyllum xanthoxylum* and *H. ammodendron* could be used as osmoregulation for osmotic adjustment under drought stress [13,19]. K^+^ is a vital regulator for cellular metabolism, including protein synthesis, enzyme activation and osmotic regulation, and maintaining of K^+^ homeostasis is necessary for plant tolerance to environmental stress [75]. Also, it was demonstrated that Ca^2+^ is responsible for regulating plant growth, development and vital signal transduction triggered by various environmental stresses [26,28]. In our study, the level of Na^+^ increased significantly in shoots of *H. ammodendron* with the duration of sorbitol treatments, which is in accordance with dynamic transcript profile of the up-regulation of genes for *CNGC*s; the level of K^+^ increased slightly in accordance with the up-regulation of genes for *AKT1*; the concentration of Ca^2+^ in tissue has no significant alteration, maintaining the stability of internal environment for signal transduction under stress conditions, which is in accordance with the up-regulation of genes for Ca^2+^-ATPase and down-regulation of genes for Ca^2+^/H^+^ antiporter and V-H^+^-PPase.

Proline, as an important osmoprotectant in plants, could maintain cell turgor and function in osmotic adjustment, playing pivotal roles in many plants responding to a wide range of environmental stimuli [62]. In our study, the level of free proline increased with the duration of sorbitol treatments, which is in accordance with the up-regulation of genes for delta-1-pyrroline-5-carboxylate synthase related to proline synthesis. Glycine betaine was considered as one of the most efficient osmoprotectants in many plants [76]. It proved that the accumulation of betaine was improved in the naturally accumulating plants exposed to variously environmental stresses [77], and plants possessing higher levels of betaine exhibited enhanced tolerance to stressful conditions [78]. In the present study, the level of betaine increased significantly with the duration of sorbitol treatments, which is in accordance with the up-regulation of genes for l-allo-threonine aldolase-like and phosphoethanolamine *N*-methyltransferase related to betaine synthesis. Soluble sugars were important metabolic resources and structural constituents of plant cells and regulate various processes, especially interacting with stress pathways to modulate plant metabolic responses [79]. It was reported that soluble sugars were accumulated in different plant parts when subjected to drought stress [80]. In the present study, the level of soluble sugars increased dramatically with the duration of sorbitol treatments, which is in accordance with the differential expression of genes related to sucrose synthesis.

Drought lead to osmotic stress, causing osmotic imbalance [81]. The accumulation of compatible osmolytes, including soluble sugars, proline, glutamic acid and glycine betaine, are very useful for plant osmotic adjustment and maintaining a stable intracellular environment in response to abiotic stress [80,81]. In the present study, the osmotic potential decreased with the duration of sorbitol treatments, which is in accordance with both the physiological data and the up-regulation of genes for inorganic ions including Na^+^ and K^+^, and organic solutes including proline, betaine and soluble sugar.

POD was a crucial antioxidant enzyme involved in ROS scavenging [19] and POD coupled with thioredoxin was found to scavenge H_2_O_2_ [41]. In the present study, the activity of POD increased significantly with the duration of sorbitol treatments, which is in accordance with the up-regulation of genes encoding peroxidase.

## 4. Materials and Methods

### 4.1. Plant Materials and Treatment Conditions

Seeds of *Haloxylon ammodendron*, collected from Alxa League, Inner Mongolia, China, were surface sterilized for 5 min with 75% (*v*/*v*) ethanol solution followed by 5 times of rinsing with sterile water and then placed on the filter paper, with 25 ± 2 °C in the dark for 12 h. After germination, uniform seedlings were transplanted to plugged holes (5 cm × 5 cm × 5 cm; one seedling/hole) in plastic containers containing heat-sterilized vermiculite and 1/2 Hoagland nutrient solution for growth in a greenhouse with temperature of 28 ± 2 °C/23 ± 2 °C (day/night), the daily photoperiod of 16 h/d, the flux density of 1200 µmol m^−2^s^−1^ and the relative humidity of 50–70%. Solutions were changed once every three days to maintain constant nutrient concentrations. 

Eight-week-old seedlings were treated with 1/2 Hoagland nutrient solution supplemented with 0.4% sorbitol to maintain the osmotic potential as −0.75 MPa or no sorbitol as control. Shoot and root samples were harvested 0, 6 h and 24 h after treatments (named as S-0, S-6, S-24, R-0, R-6 and R-24, respectively) and frozen in liquid nitrogen and stored at −80 °C for RNA-seq, qRT-PCR and relevant physiological analyses.

### 4.2. RNA Extraction, cDNA Library Creation, and Sequencing

Total RNA was extracted three times from the six samples S-0, S-6, S-24, R-0, R-6 and R-24, respectively, using the TRIzol reagent (Invitrogen, Carlsbad, CA, USA). Equivalent amount of total RNA isolated from shoots and roots was pooled, individually, and the two mRNA pools were used to create the final two cDNA library in shoots and root, and then sequenced on the Illumina HiSeq™2000 platform in BGI Shenzhen, respectively. All sequencing datasets are available in the NCBI database with the accession number GSE93684 (https://www.ncbi.nlm.nih.gov/geo/query/acc.cgi?acc=GSE93684).

### 4.3. De Novo Transcriptome Assembly and Functional Annotation

Reads from each library were assembled separately [15]. Then the Trinity unigenes of both two libraries were clustered with TGICL software to get sequences that cannot be extended on either end [82]. After performing gene family clustering, the unigenes were divided into two classes. One was cluster, of which the prefix was CL and contains several unigenes that shared above 70% sequence similarity to each other, and the other was singletons with the prefix of unigenes.

Functional annotation could provide information on expression and function of unigenes. We annotated the sequences based on protein databases including Nr, Nt (non-redundant nucleotide sequences), Swiss-Prot, KEGG, COG and GO (E-value < 10^−5^) by retrieving the proteins with the highest sequence similarity to *H. ammodendron* unigenes. The Blast2GO program was employed to obtain GO annotations and WEGO software was used to perform GO functional classification of all unigenes [83].

### 4.4. DGE Library Preparation and Sequencing

Six independent Tag library preparation for the different time points of shoots and roots after sorbitol treatment (S-0, S-6, S-24, R-0, R-6 and R-24) were performed in parallel according to the protocol of a tag-based DGE system [84]. Then each library was sequenced using the Illumina HiSeq™2000 sequencing platform, and the reads were mapped to the transcriptome reference database. For gene expression analysis, the number of clean tags for each gene was calculated and normalized to the number of TPM tags [84]. Both false discovery rate (FDR) ≤0.005 and an absolute value of the log2 ratio ≥1 were used as the threshold to determine DEGs [84].

### 4.5. qRT-PCR Validation of Gene Expressions

Ten candidate annotated genes were randomly selected for validation of differential expression through quantitative reverse transcription polymerase chain reaction (qRT-PCR). Total RNA was extracted from three replicated biological samples individually for each treatment of shoots and roots. qRT-PCR was conducted using SYBR Green dye (SYBR^®^ Green Real-time PCR Master Mix-Plus, Code No. QPK-212) and performed on the ABI StepOnePlus Real-Time PCR System. The relative expression levels of genes were calculated using the 2^−ΔΔ*C*t^ method. *ACTIN* was used as the housekeeping gene. The fold changes of both relative gene expression levels by qRT-PCR and their DGE profilings were calculated and their linear regression analysis was conducted.

### 4.6. Measurement of Physiological Parameters

Leaf osmotic potential, soluble sugar, POD activity, free proline and betaine were measured [85], respectively. There were six replications with 15 seedlings bound together as one replication for each analysis.

## 5. Conclusions

The gene expression changes at the transcriptional scale in *H. ammodendron* under −0.75 MPa osmotic stress were profiled, and abundance of genes involved in ion transport, signal transduction, ROS scavenging, photosynthesis, cell wall organization, membrane stabilization, secondary metabolism and hormones were significantly increased. Especially, the physiological changes of inorganic ions and organic solutes were in accordance with the dynamic transcript profile. A detailed investigation of the pathways and candidate genes identified in this study promote the research on the molecular mechanisms of abiotic stress tolerance in the xero-halophytic species, and lay a solid foundation for developing stress-tolerant forage and crop species by using excellent gene resources from *H. ammodendron*.

## Figures and Tables

**Table 1:** Overview of de novo assembly of transcriptome sequence.

Unigenes	Total Number	Total Length (bp)	Mean Length (bp)	N50 (bp)
Shoots	82,736	46,038,066	556	867
Roots	99,624	53,273,063	535	828
All	87,109	59,244,982	680	1064

## Supplementary Materials

Supplementary materials can be found at www.mdpi.com/1422-0067/19/1/84/s1.

## Abbreviations

ABA	Abscisic acid
AKT	*Arabidopsis* K^+^ transporter
BP	Biological process
C4H	Cinnamate 4-hydroxylase
CC	Cellular component
CNGC	Cyclic nucleotide-gated channel
COG	Clusters of orthologous groups of protein
CV	Coefficient of variation
CYP450	Cytochrome P450
DEGs	Differentially expressed genes
DGE	Digital gene expression
FBA	Fructose-1,6-bisphosphate aldolase
FDR	False discovery rate
GO	Gene ontology
JA	Jasmonic acid
KEGG	Kyoto encyclopedia of genes and genomes
LEA	Late embryogenesis abundant protein
MF	Molecular function
Nr	Non-redundant protein database
Nt	Non-redundant nucleotide sequences
POD	Peroxidase
qRT-PCR	Quantitative reverse transcription polymerase chain reaction
RLK	Receptor-like protein kinase
ROS	Reactive oxygen species
sHSP	small heat-shock protein
SSRs	Simple sequence repeats
TF	Transcription factor
TPM	Transcripts per million

## References

[1] Zou J.J., Li X.D., Ratnasekera D., Wang C., Liu W.X., Song L.F., Zhang W.Z., Wu W.H. (2015). *Arabidopsis* CALCIUM-DEPENDENT PROTEIN KINASE8 and CATALASE3 Function in Abscisic Acid-Mediated Signaling and H_2_O_2_ Homeostasis in Stomatal Guard Cells under Drought Stress. Plant Cell.

[2] Farooq M., Wahid A., Kobayashi N., Fujita D., Basra S.M.A. (2009). Plant Drought Stress: Effects, mechanisms and management. Agron. Sustain. Dev..

[3] Zhou Y.J., Gao F., Liu R., Feng J.C., Li H.J. (2012). De novo sequencing and analysis of root transcriptome using 454 pyrosequencing to discover putative genes associated with drought tolerance in *Ammopiptanthus mongolicus*. BMC Genom..

[4] Pangle R.E., Limousin J.M., Plaut J.A., Pockman W., McDowell N.G. (2015). Prolonged experimental drought reduces plant hydraulic conductance and transpiration and increases mortality in a pinon–Atriplex woodland. Ecol. Evol..

[5] Ashraf M. (2010). Inducing drought tolerance in plants: Recent advances. Biotechnol. Adv..

[6] Wu G., Xi J., Wang Q., Bao A., Ma Q., Zhang J., Wang S.M. (2011). The *ZxNHX* gene encoding tonoplast Na^+^/H^+^ antiporter from the xerophyte *Zygophyllum xanthoxylum* plays important roles in response to salt and drought. J. Plant Physiol..

[7] Dong X., Zhang X. (2001). Some observations of the adaptations of sandy shrubs to the arid environment in the Mu Us Sandland: Leaf water relations and anatomic features. J. Arid Environ..

[8] Yang W.B., Feng W., Jia Z.Q., Zhu Y.J., Guo J.Y. (2014). Soil water threshold for the growth of *Haloxylon ammodendron* in the Ulan Buh desert in arid northwest China. S. Afr. J. Bot..

[9] Liu J.L., Wang Y.G., Yang X.H., Wang B.F. (2010). Genetic variation in seed and seedling traits of six *Haloxylon ammodendron* shrub provenances in desert areas of China. Agroforest. Syst..

[10] Kang J.J., Zhao W.Z., Su P.X., Yang Z.H. (2014). Sodium (Na^+^) and silicon (Si) coexistence promotes growth and enhances drought resistance of the succulent xerophyte *Haloxylon ammodendron*. Soil Sci. Plant Nutr..

[11] Guo Q., Tan D., Liu Y., Wang C. (2004). Advance in studies of *Haloxylon bunge*’s mechanism of adapation and resistance to drought. Forest Res..

[12] Wang S., Wan C., Wang Y., Chen H., Zhou Z., Fu H., Sosebee R.E. (2004). The characteristics of Na^+^, K^+^ and free proline distribution in several drought-resistant plants of the Alxa Desert, China. J. Arid Environ..

[13] Wang Z., Gerstein M., Snyder M. (2009). RNA-Seq: A revolutionary tool for Transcriptomics. Nat. Rev. Genet..

[14] Qiu Q., Ma T., Hu Q.J., Liu B.B., Wu Y.X., Zhou H.H., Wang Q., Wang J., Liu J.Q. (2011). Genome-scale transcriptome analysis of the desert poplar, *Populus euphratica*. Tree Physiol..

[15] Ma T., Wang J.Y., Zhou G.K., Yue Z., Wang J., Liu J.Q. (2013). Genomic insights into salt adaptation in a desert poplar. Nat. Commun..

[16] Xiao L., Yang G., Zhu J.K., Oliver M.J., He Y. (2015). The resurrection genome of *Boea hygrometrica*: A blueprint for survival of dehydration. Proc. Natl. Acad. Sci. USA.

[17] Gugger P.F., Peñaloza-Ramírez J.M., Wright J.W., Sork V.L. (2016). Whole-transcriptome response to water stress in a California endemic oak, *Quercus lobata*. Tree Physiol..

[18] Jia J.B., Zhou J., Shi W.G., Cao X., Luo J., Polle A., Luo Z.B. (2017). Comparative transcriptomic analysis reveals the roles of overlapping heat-/drought responsive genes in poplars exposed to high temperature and drought. Sci. Rep..

[19] Ma Q., Bao A.K., Chai W.W., Wang W.Y., Zhang J.L., Li Y.X., Wang S.W. (2016). Transcriptomic analysis of the succulent xerophyte *Zygophyllum xanthoxylum* in response to salt treatment and osmotic stress. Plant Soil.

[20] Jin Y., Jing W., Zhang Q., Zhang W. (2015). Cyclic nucleotide gated channel 10 negatively regulates salt tolerance by mediating Na^+^ transport in *Arabidopsis*. J. Plant Res..

[21] Wang S.M., Zhang J.L., Flowers T.J. (2007). Low-affinity Na^+^ uptake in the *halophyte Suaeda maritima*. Plant Physiol..

[22] Qudeimat E., Faltusz A.M.C., Wheeler G., Lang D., Brownlee C., Reski R., Frank W. (2008). A PIIB-type Ca^2+^ATPase is essential for stress adaptation in *Physcomitrella patens*. Proc. Natl. Acad. Sci. USA.

[23] Manohar M., Shigaki T., Hirschi K.D. (2011). Plant cation/H^+^ exchangers (CAXs): Biological functions and genetic manipulations. Plant Biol..

[24] Gaxiola R.A., Palmgren M.G., Schumacher K. (2007). Plant proton pumps. FEBS Lett..

[25] Yu S.C., Zhang F.L., Yu Y.J., Zhang D.S., Zhao X.Y. (2012). Transcriptome Profiling of Dehydration Stress in the Chinese Cabbage (*Brassica rapa* L. ssp. pekinensis) by Tag Sequencing. Plant Mol. Biol. Rep..

[26] Bai Z.Y., Wang T., Wu Y.H., Wang K., Liang Q.Y., Pan Y.Z. (2017). Whole-transcriptome sequence analysis of differentially expressed genes in *Phormium tenaxunder* drought stress. Sci. Rep..

[27] Kang M., Fokar M., Abdelmageed H., Allen R.D. (2011). *Arabidopsis* SAP5 functions as a positive regulator of stress responses and exhibits E3 ubiquitin ligase activity. Plant Mol. Biol..

[28] Reddy A.S., Ali G.S., Celesnik H., Day I.S. (2011). Coping with stresses: Roles of calcium- and calcium/calmodulin-regulated gene expression. Plant Cell.

[29] Ichimura K., Shinozaki K., Tena G., Sheen J., Henry Y., Heberle-Bors E. (2002). Mitogen-activated protein kinase cascades in plants: A new nomenclature. Trends Plant Sci..

[30] Vlad F., Rubio S., Rodrigues A., Sirichandra C., Belin C., Robert N., Leung J., Rodriguez P.L., Laurière C., Merlot S. (2009). Protein phosphatases 2C regulate the activation of the Snf1-related kinase OST1 by abscisic acid in *Arabidopsis*. Plant Cell.

[31] Nambara E., Marion-Poll A. (2005). Abscisic acid biosynthesis and catabolism. Annu. Rev. Plant Biol..

[32] Martin R.C., Mok D.W.S., Smets R., van Onckelen H.A., Mok M.C. (2001). Development of transgenic tobacco harboring a zeatin *O*-glucosyl transferase gene from *phaseolus*. In Vitro Cell Dev. Plant.

[33] Martinelli F., Uratsu S.L., Albrecht U., Reagan R.L., Phu M.L., Britton M., Buffalo V., Fass J., Leicht E., Zhao W.X. (2012). Transcriptome profiling of citrus fruit response to huanglongbing disease. PLoS ONE.

[34] Wang P., Yang C., Chen S., Zhang X., Wang D.J. (2017). Transcriptomic basis for drought resistance in *Brassica napus* L.. Sci. Rep..

[35] Wang H., Liang X., Wan Q., Wang X., Bi Y. (2009). Ethylene and nitric oxide are involved in maintaining ion homeostasis in *Arabidopsis* callus under salt stress. Planta.

[36] Cenzano A., Abdala G., Hause B. (2007). Cytochemical immuno-localization of allene oxide cyclase, a jasmonic acid biosynthetic enzyme, in developing potato stolons. J. Plant Physiol..

[37] Tani T., Sobajima H., Okada K., Chujo T., Arimura S.I., Tsutsumi N., Nishimura M., Seto H., Nojiri H., Yamane H. (2007). Identification of the *OsOPR7* gene encoding 12-oxophytodienoate reductase involved in the biosynthesis of jasmonic acid in rice. Planta.

[38] Shan C., Liang Z. (2010). Jasmonic acid regulates ascorbate and glutathione metabolism in *Agropyron cristatum* leaves under water stress. Plant Sci..

[39] Zalewski W., Galuszka P., Gasparis S., Orczyk W., Nadolska-Orczyk A. (2010). Silencing of the *HvCKX1* gene decreases the cytokinin oxidase/dehydrogenase level in barley and leads to higher plant productivity. J. Exp. Bot..

[40] Reguera M., Peleg Z., Abdel-Tawab Y.M., Tumimbang E.B., Delatorre C.A., Blumwald E. (2013). Stress-induced cytokinin synthesis increases drought tolerance through the coordinated regulation of carbon and nitrogen assimilation in rice. Plant Physiol..

[41] Sevilla F., Camejo D., Ortiz-Espin A., Calderon A., Lazaro J.J., Jimenez A. (2015). The thioredoxin/peroxiredoxin/sulfiredoxin system: Current overview on its redox function in plants and regulation by reactive oxygen and nitrogen species. J. Exp. Bot..

[42] Dahal K., Vanlerberghe G.C. (2017). Alternative oxidase respiration maintains both mitochondrial and chloroplast function during drought. New Phytol..

[43] Cho S.K., Kim J.E., Park J.A., Eom T.J., Kim W.T. (2006). Constitutive expression of abiotic stress-inducible hot pepper *CaXTH3*, which encodes a xyloglucan endotransglucosylase/hydrolase homolog, improves drought and salt tolerance in transgenic *Arabidopsis* plants. FEBS Lett..

[44] Li C.Y., Deng G.M., Yang J., Viljoen A., Jin Y., Kuang R.B., Zuo C.W., Lv Z.C., Yang Q.S., Sheng O. (2012). Transcriptome profiling of resistant and susceptible Cavendish banana roots following inoculation with *Fusarium oxysporum* f. sp. *cubense* tropical race 4. BMC Genom..

[45] Bray E.A. (2004). Genes commonly regulated by water-deficit stress in *Arabidopsis thaliana*. J. Exp. Bot..

[46] Zheng J., Fu J., Gou M., Huai J., Liu Y., Jian M., Huang Q.S., Guo X.Y., Dong Z.G., Wang H.Z. (2010). Genome-wide transcriptome analysis of two maize inbred lines under drought stress. Plant Mol. Biol..

[47] Weng J., Chapple C. (2010). The origin and evolution of lignin biosynthesis. New Phytol..

[48] Liang M., Haroldsen V., Cai X., Wu Y. (2006). Expression of a putative laccase gene, *ZmLAC1*, in maize primary roots under stress. Plant Cell Environ..

[49] Bjurhager I., Olsson A., Zhang B., Gerber L.R., Kumar M.S., Berglund L.A., Burgert I., Sundberg B., Salme´n L. (2010). Ultrastructure and mechanical properties of populus wood with reduced lignin content caused by transgenic down-regulation of cinnamate 4-hydroxylase. Biomacromolecules.

[50] Duan J., Zhang M., Zhang H., Xiong H., Liu P., Ali J., Li J.J., Li Z.C. (2012). *OsMIOX*, a myo-inositol oxygenase gene, improves drought tolerance through scavenging of reactive oxygen species in rice (*Oryza sativa* L.). Plant Sci..

[51] Li N., Xu C., Libeisson Y., Philippar K. (2016). Fatty acid and lipid transport in plant cells. Trends Plant Sci..

[52] Dao T.T.H., Linthorst H.J.M., Verpoorte R. (2011). Chalcone synthase and its functions in plant resistance. Phytochem. Rev..

[53] Dehghan S., Sadeghi M., Poppel A., Fischer R., Lakes-Harlan R., Kavousi H.R. (2014). Differential inductions of phenylalanine ammonia-lyase and chalcone synthase during wounding, salicylic acid treatment, and salinity stress in safflower, *Carthamus tinctorius*. Biosci. Rep..

[54] Abrahams S., Lee E., Walker A.R., Tanner G.J., Larkin P.J., Ashton A.R. (2003). The *Arabidopsis TDS4* gene encodes leucoanthocyanidin dioxygenase (LDOX) and is essential for proanthocyanidin synthesis and vacuole development. Plant J..

[55] Harish M.C., Dachinamoorthy P., Balamurugan S., Bala Murugan S., Sathishkumar R. (2013). Overexpression of homogentisate phytyltransferase (HPT) and tocopherol cyclase (TC) enhances α-tocopherol content in transgenic tobacco. Biol. Plantarum.

[56] Nebert D.W., Dalton T.P. (2006). The role of cytochrome P450 enzymes in endogenous signaling pathways and environmental carcinogenesis. Nat. Rev. Cancer.

[57] Pan Y., Michael T.P., Hudson M.E., Kay S.A., Chory J., Schuler M.A. (2009). Cytochrome P450 monooxygenases as reporters for circadian-regulated pathways. Plant Physiol..

[58] Kurepin L.V., Ivanov A.G., Zaman M., Pharis R.P., Allakhverdiev S.I., Hurry V. (2015). Stress-related hormones and glycinebetaine interplay in protection of photosynthesis under abiotic stress conditions. Photosynth. Res..

[59] Diray-Arce J., Clement M., Gul B., Khan M.A., Nielsen B.L. (2015). Transcriptome assembly, profiling and differential gene expression analysis of the halophyte *Suaeda fruticosa* provides insights into salt tolerance. BMC Genom..

[60] Liu X., Wang Z., Wang L., Wu R., Phillips J., Deng X. (2009). LEA 4 group genes from the resurrection plant *Boea hygrometrica* confer dehydration tolerance in transgenic tobacco. Plant Sci..

[61] Kosova K., Vitamvas P., Prasil I.T. (2014). Wheat and barley dehydrins under cold, drought, and salinity—What can LEA-II proteins tell us about plant stress response?. Front. Plant Sci..

[62] Stein H., Honig A., Miller G., Erster O., Eilenberg H., Csonka L.N., Szabados L., Koncz C., Zilberstein A. (2011). Elevation of free proline and proline-rich protein levels by simultaneous manipulations of proline biosynthesis and degradation in plants. Plant Sci..

[63] Malinovska L., Kroschwald S., Munder M.C., Richter D., Alberti S. (2012). Molecular chaperones and stress-inducible protein-sorting factors coordinate the spatiotemporal distribution of protein aggregates. Mol. Biol. Cell.

[64] Sato Y., Yokoya S. (2008). Enhanced tolerance to drought stress in transgenic rice plants overexpressing a small heat-shock protein, sHSP17.7. Plant Cell Rep..

[65] Dana M.D.L.M., Pintor-Toro J.A., Cubero B. (2006). Transgenic tobacco plants overexpressing chitinases of fungal origin show enhanced resistance to biotic and abiotic stress agents. Plant Physiol..

[66] Liu L., Yang J.Y., Yan S.J., Zhang S.H., Zhu X.Y., Liu B. (2016). The germin-like protein OsGLP2-1 enhances resistance to fungal blast and bacterial blight in rice. Plant Mol. Biol..

[67] Gokulakannan G.G., Niehaus K. (2010). Characterization of the Medicago truncatula cell wall proteome in cell suspension culture upon elicitation and suppression of plant defense. J. Plant Physiol..

[68] Chaves M.M., Flexas J., Pinheiro C. (2009). Photosynthesis under drought and salt stress: Regulation mechanisms from whole plant to cell. Ann. Bot..

[69] Li C.L., Wang Y., Liu L., Hu Y., Zhang F., Mergen S., Wang G.D., Schläppi M.R., Chu C.C. (2011). A rice plastidial nucleotide sugar epimerase is involved in galactolipid biosynthesis and improves photosynthetic efficiency. PLoS Genet..

[70] Ma W., Wei L., Wang Q., Shi D., Chen H. (2006). Increased activity of the non-regulated enzymes fructose-1,6-bisphosphate aldolase and triosephosphate isomerase in *Anabaena* sp. strain PCC 7120 increases photosynthetic yield. J. Appl. Phycol..

[71] Woodson J.D., Perezruiz J.M., Chory J. (2011). Heme synthesis by plastid ferrochelatase I regulates nuclear gene expression in Plants. Curr. Biol..

[72] David P., des Francssmall C.C., Sevignac M., Thareau V., Macadre C., Langin T., Geffroy V. (2010). Three highly similar formate dehydrogenase genes located in the vicinity of the B4 resistance gene cluster are differentially expressed under biotic and abiotic stresses in *Phaseolus vulgaris*. Theor. Appl. Genet..

[73] Guo L., Devaiah S.P., Narasimhan R., Pan X.Q., Zhang Y.Y., Zhang W.H. (2012). Cytosolic glyceraldehyde-3-phosphate dehydrogenases interact with phospholipase D-δ to transduce hydrogen peroxide signals in the *Arabidopsis* response to stress. Plant Cell.

[74] Zhang J.L., Flowers T.J., Wang S.M. (2010). Mechanisms of sodium uptake by roots of higher plants. Plant Soil.

[75] Anschütz U., Becker D., Shabala S. (2014). Going beyond nutrition: Regulation of potassium homoeostasis as a common denominator of plant adaptive responses to environment. J. Plant Physiol..

[76] Waditee R., Bhuiyan N.H., Hirata E., Hibino T., Tanaka Y., Shikata M., Takabe T. (2007). Metabolic engineering for betaine accumulation in microbes and plants. J. Biol. Chem..

[77] Chen T.H.H., Murata N. (2011). Glycinebetaine protects plants against abiotic stress: Mechanisms and biotechnological applications. Plant Cell Environ..

[78] Chen T.H.H., Murata N. (2008). Glycinebetaine: An effective protectant against abiotic stress in plants. Trends Plant Sci..

[79] Loreti E., Bellis L.D., Alpi A., Perata P. (2001). Why and how do plant cells sense sugars?. Ann. Bot..

[80] Hu H.H., Dai M.Q., Yao J.L., Xiao B.Z., Li X.G., Zhang Q.F., Xiong L.Z. (2006). Overexpressing a NAM, ATAF, and CUC (NAC) transcription factor enhances drought resistance and salt tolerance in rice. Proc. Natl. Acad. Sci. USA.

[81] Wang S., Liang D., Li C., Hao Y., Ma F., Shu H. (2012). Influence of drought stress on the cellular ultrastructure and antioxidant system in leaves of drought-tolerant and drought-sensitive apple rootstocks. Plant Physiol. Biochem..

[82] Pertea G., Huang X., Liang F., Antonescu V., Sultana R., Karamycheva S., Lee Y., White J., Cheung F., Quackenbush J. (2003). TIGR Gene Indices clustering tools (TGICL): A software system for fast clustering of large EST datasets. Bioinformatics.

[83] Ye J., Fang L., Zheng H., Zhang Y., Chen J., Zhang Z., Wang J., Li S., Li R., Bolund L. (2006). WEGO: A web tool for plotting GO annotations. Nucleic Acids Res..

[84] Xue J., Bao Y.Y., Li B.-L., Cheng Y.B., Peng Z.Y., Liu H., Xu H.J., Zhu Z.R., Lou Y.G., Cheng J.A., Zhang C.X. (2010). Transcriptome analysis of the brown planthopper *Nilaparvata lugens*. PLoS ONE.

[85] Liu H., Yu C.Y., Li H.X., Ouyang B., Wang T.T., Zhang J.H. (2015). Overexpression of *ShDHN*, a dehydrin gene from *Solanum habrochaites* enhances tolerance to multiple abiotic stresses in tomato. Plant Sci..

