# Supplementary material for: Transcriptomic Profiling and Physiological Analysis of Haloxylon ammodendron in Response to Osmotic Stress
Source: Int J Mol Sci. 2017 Dec 29;19(1):84. doi: 10.3390/ijms19010084 (PMC5796034; doi:10.3390/ijms19010084)
Supplement: Supplementary file 1 [file ijms-19-00084-s001.pdf]

Supplementary Materials: Transcriptomic profiling and physiological analysis of *Haloxylon ammodendron* in response to osmotic stress

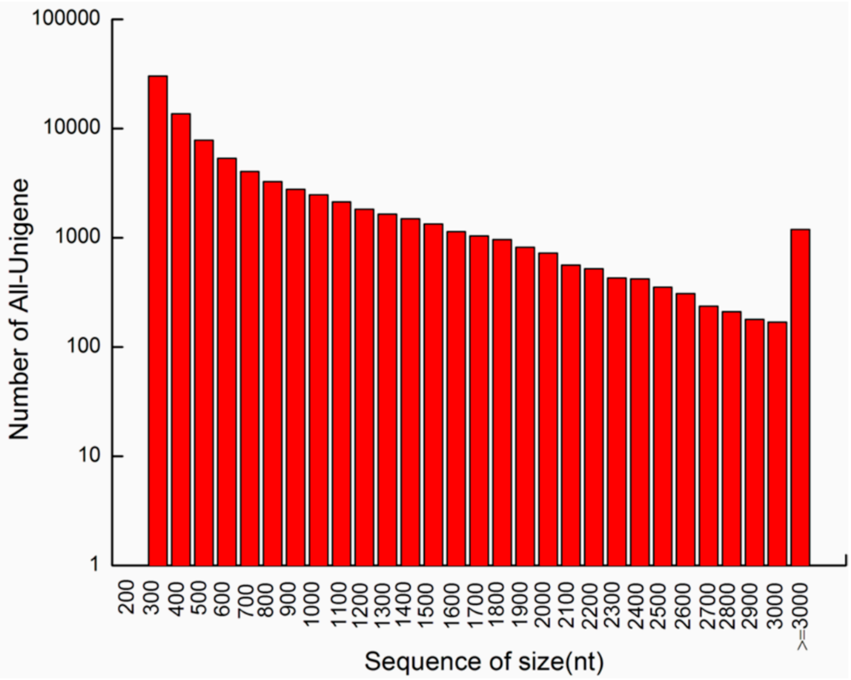

Figure S1. Length distribution of assembled Unigenes.

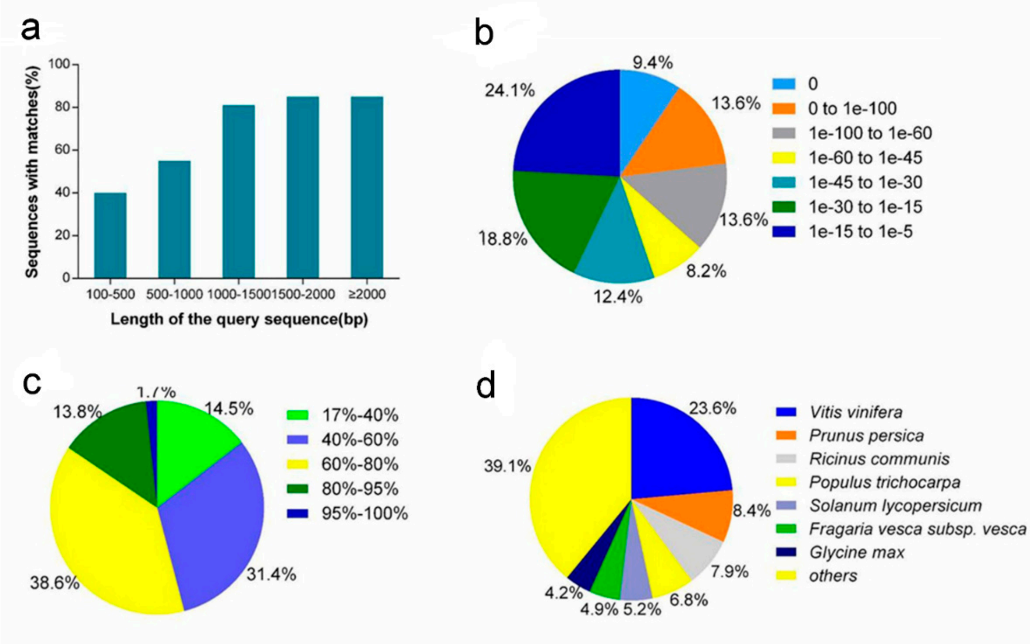

Figure S2. Characteristics of homology search of query sequences aligned by BLASTx to the nr database. (a) Effect of query length on the BLAST matches. (b) E-value distribution of unigenes BLASTx hits in the nr database with an E-value threshold of 1.0E-5. (c) Species distribution of the top BLAST hits for each sequence with a cut-off E-value of 1.0E-5. (d) Similarity distribution of the top BLAST hits for each sequence with a cut-off E-value of 1.0E-5.

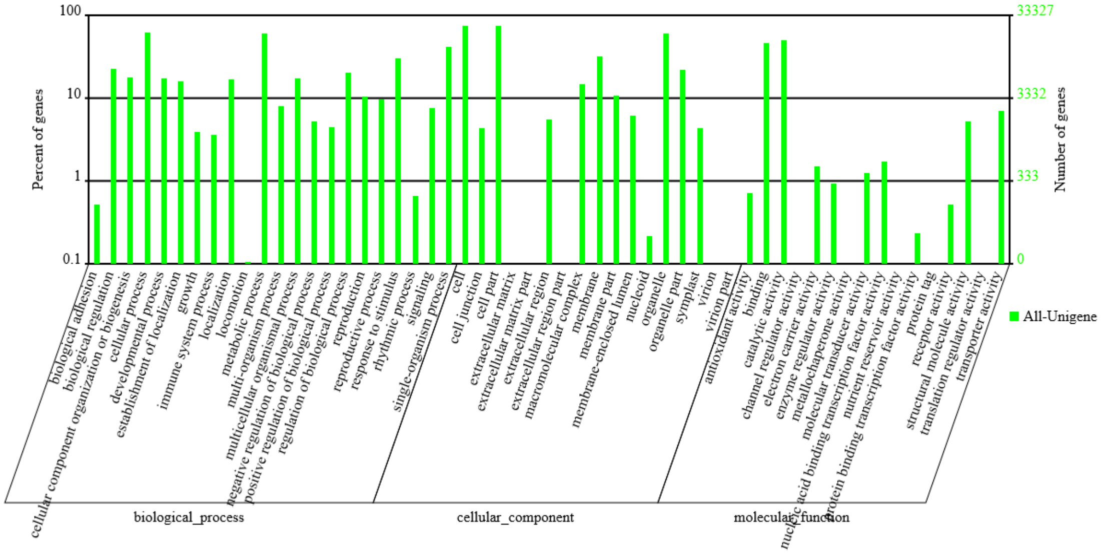

Figure S3. Functional classifications of GO terms of all assembled unigenes. The results are summarized in three main categories: biological process, cellular component, and molecular function. The right y-axis indicates the number of genes in a category. The left y-axis indicates percentage of a specific category of genes.

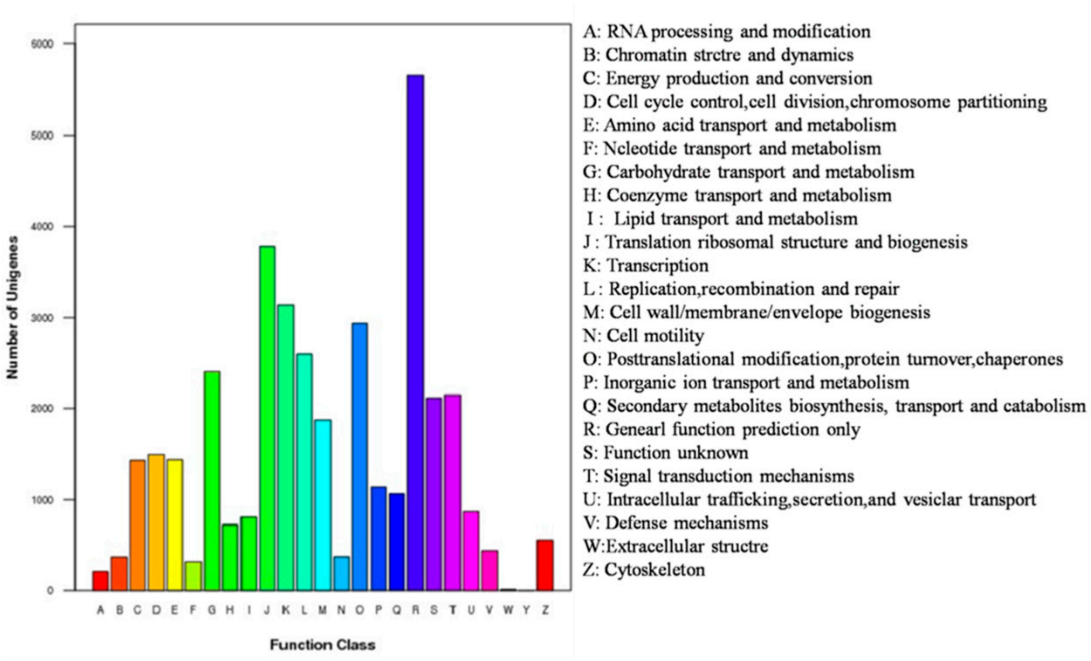

Figure S4. Distribution of unigenes in the transcriptome with COG functional classification. 19196 sequences have a COG classification among 25 categories.

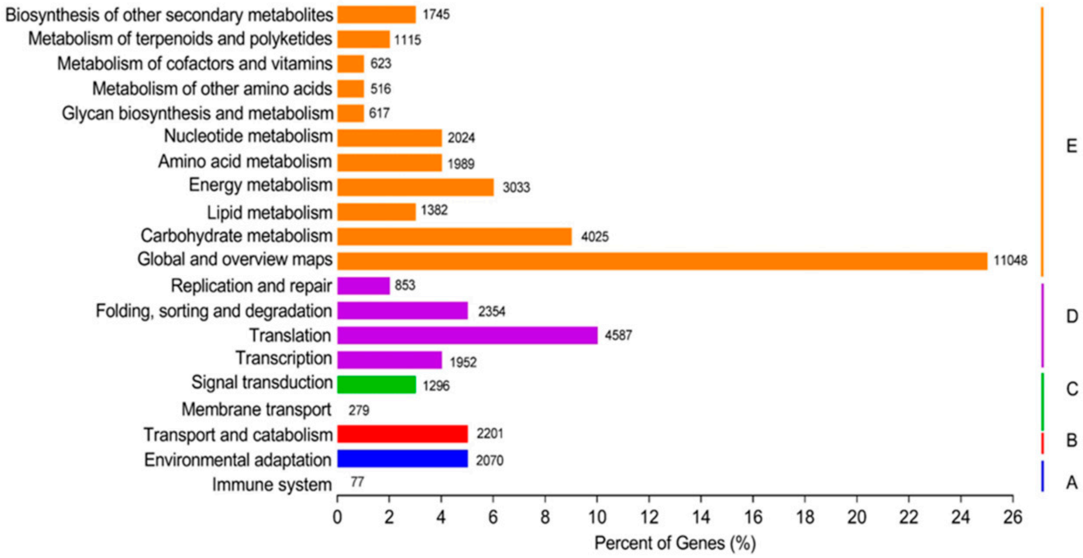

Figure S5. Number of all-unigenes in each clade of the KEGG pathway maps. The all-unigenes were classified into 20 clades under five major categories. In each category, the clades are listed according to their abundancies of all-unigenes.

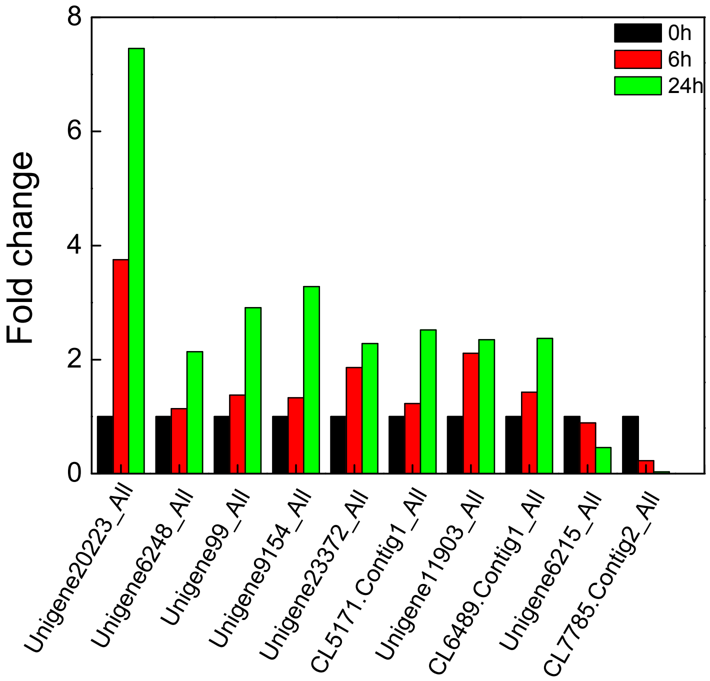

Figure S6. The fold changes of relative gene expression levels by Quantitative RT-PCR analysis for ten randomly selected DEGs.

Table S1 Output of statistics of sequence analysis

Samples	Total raw reads	Total clean reads	Total clean nucleotides(nt)	Q20 percentage	N percentage	GC percentage
Shoots	76,087,174	69,414,094	6,247,268,460	98.04%	0.00%	43.50%
Roots	75,376,466	69,232,936	6,230,964,240	97.90%	0.00%	43.95%

Table S2 BLAST analysis of the non-redundant unigenes against public databases

Database	Nr	Nt	Swiss-prot	KEGG	COG	GO	All
Hit numbers	47,522	32,356	31,797	28,794	19,196	33,327	50,452
%	54.55	37.14	36.50	33.05	22.04	38.26	57.92

Table S3 Statistics of DGE sequencing of shoots and leaves of *H. ammodendron on* under drought stress

Summary	S-0	S-6	S-24	R-0	R-6	R-24
Raw Tags	11,780,059	11,733,897	12,256,083	11,771,062	12,106,534	12,490,186
Distinct Tags	1,181,245	1,230,050	1,481,336	1,490,453	1,566,343	1,782,339
Clean Tags	11,120,936	11,073,647	11476269	10,908,694	11,240,442	11,520,622
Distinct Clean Tags	528,477	575,636	706493	633,042	705,321	817,454
Map to Gene Tags	8,445,700 (75.94%)	8,093,382 (73.09%)	7773532 (67.74%)	7,561,770 (69.32%)	7,307,256 (65.01%)	6,961,351 (60.43%)
Unique Map to Gene Tags	7,095,240 (63.80%)	6,779,012 (61.22%)	6506445 (56.69%)	6,399,320 (58.66%)	6,114,393 (54.40%)	5,755,489 (49.96%)

Table S4 The continuously up-regulated or down-regulated unigenes related to transporters in shoots and roots in *H. ammodendron* under sorbitol treatment

Gene ID	Log2 ratio		Functional annotation
	6h/0h	24h/0h	
Shoots			
Unigene99_All	0.59	1.30	AKT1-like
Unigene19843_All	0.59	1.90	Mkt1p
CL4885.Contig2_All	0.26	1.05	CNGC-17-like
Unigene6248_All	0.34	1.36	ER-type calcium ATPase
Unigene2055_All	-1.03	-1.00	calcium antiporter 1
Unigene20156_All	-0.55	-1.22	vacuolar H^+-pyrophosphatase
Roots			
Unigene23862_All	-1.04	-3.92	vacuolar iron transporter
Unigene11712_All	-2.48	-3.13	nitrate transporter 1.1
Unigene12228_All	-0.35	-1.82	cation/H^+ antiporter 20-like

Table S5 The continuously up-regulated or down-regulated unigenes related to signal transduction in shoots and roots in *H. ammodendron* under sorbitol treatment

Gene ID	Log2 ratio		Functional annotation
	6h/0h	24h/0h	
Shoots			
Unigene11736_All	3.91	5.36	WRKY6
Unigene13756_All	0.75	1.99	WRKY1
CL1607.Contig1_All	1.66	2.60	WRKY1
Unigene25241_All	1.33	2.00	WRKY

Unigene1029_All	1.49	1.67	Myb14
Unigene23372_All	0.14	1.40	MYB39-like
Unigene2036_All	1.97	3.10	erf8
Unigene21677_All	1.22	3.65	NAC
CL5088.Contig3_All	1.35	3.52	AP2
CL5171.Contig1_All	0.68	2.35	GRAS
Unigene3111_All	0.41	2.88	AP2/ERF
Unigene11527_All	0.52	2.17	AP2/ERF
Unigene10170_All	0.37	1.80	ERF11
Unigene12334_All	0.73	1.39	HSF24-like
Unigene16628_All	1.20	2.55	ERF9-like
Unigene4961_All	2.12	2.27	Homeobox leucine zipper protein
Unigene3770_All	0.93	1.62	CCCH-type zinc finger protein
Unigene26900_All	2.11	2.69	ZAT10-like
CL6105.Contig2_All	1.87	2.95	Calmodulin-binding protein
Unigene917_All	0.37	1.81	Putative calcium-binding protein
CL7251.Contig1_All	0.13	1.74	Calcium-binding protein CML42
Unigene868_All	2.47	4.26	MAPKK-2
CL3509.Contig2_All	0.10	2.09	MAPKK-2
CL7538.Contig3_All	1.11	2.75	CDPK2
CL7538.Contig2_All	1.38	3.39	CDPK2
CL809.Contig3_All	1.31	1.61	CDPK10
Unigene8948_All	1.11	1.84	AMPK
Unigene978_All	0.30	1.36	Receptor protein kinase-like protein
CL1255.Contig5_All	1.36	2.07	Receptor-like protein kinase
CL2696.Contig2_All	1.19	1.91	Receptor-like protein kinase-like
Unigene34890_All	8.03	7.93	PERK1-like
Unigene56999_All	6.87	7.29	Wall-associated receptor kinase-like 1-like
Unigene10761_All	1.88	2.54	Cysteine-rich receptor-like protein kinase 10-like
Unigene11538_All	2.01	2.37	lectin S-receptor-like serine/threonine-protein kinase B120-like
Unigene5587_All	1.26	2.63	Serine/Threonine protein kinase family protein
Unigene24956_All	0.18	1.26	Serine/Threonine-protein kinase
Unigene38527_All	0.68	2.39	Serine/Threonine-protein kinase bri1
Unigene2007_All	1.58	4.57	lectin S-receptor-like serine/threonine-protein kinase
CL8172.Contig1_All	0.55	1.30	LRR receptor-like serine/threonine-protein kinase GSO1-like
Unigene6656_All	7.08	6.93	Dual-specificity protein-like phosphatase 3
Unigene8669_All	0.74	3.58	Protein phosphatase 2c
Unigene23931_All	2.20	3.15	1-Aminocyclopropane-1-carboxylate oxidase
CL10105.Contig2_All	0.39	1.44	1-Aminocyclopropane-1-carboxylate oxidase homolog
CL8424.Contig2_All	2.81	4.04	1-Aminocyclopropane-1-carboxylate synthase
Unigene19336_All	3.24	4.15	NCED
Unigene13593_All	2.95	3.43	CRT/DRE binding factor
Unigene11889_All	1.56	1.62	Phytosulfokine-alpha peptide precursor
Unigene3767_All	1.70	1.95	Phytosulfokine receptor 2-like
CL4819.Contig1_All	1.88	2.95	12-Oxophytodienoate reductase
Unigene3744_All	2.12	3.58	AOS
Unigene3327_All	2.98	3.62	Allene oxide cyclase 4, chloroplastic-like
CL2211.Contig2_All	1.97	2.39	Lipoxygenase
Unigene3018_All	5.17	6.93	Zeatin O-glucosyltransferase-like

Unigene25168_All	-0.02	1.07	Transcription factor MYC2-like
CL2902.Contig1_All	0.24	1.10	Topless-related protein 1-like
Unigene38_All	0.07	1.62	Ribose-phosphate pyrophosphokinase 2
CL3142.Contig1_All	-1.46	-1.50	Elongation factor G, chloroplastic-like
CL10135.Contig3_All	-0.72	-1.10	Eukaryotic elongation factor 1A
Unigene40486_All	0.00	-2.33	Elongation factor 1alpha
Unigene22044_All	-1.07	-2.80	bHLH51-like
CL3390.Contig1_All	-0.43	-1.07	MYB
Unigene23203_All	-0.72	-1.63	GATA
Unigene20317_All	-1.21	-1.35	Liguleless 1 protein
Unigene18371_All	-1.38	-9.59	WRKY
Unigene51144_All	-0.55	-1.79	GATA
Unigene11970_All	-1.01	-1.14	Zinc finger protein 3-like
Unigene27976_All	-0.97	-1.04	Zinc finger protein 593 homolog
Unigene23925_All	-0.64	-2.38	Zinc finger protein constans-like 16
Unigene26415_All	-0.18	-1.35	Leucine zipper protein
CL6619.Contig1_All	-0.71	-2.41	Homeobox-leucine zipper protein
Unigene26415_All	-0.18	-1.35	Leucine zipper protein
Unigene10581_All	-1.02	-1.36	Homeobox-leucine zipper protein ATHB-6-like
Unigene968_All	-0.91	-1.62	Homeobox-leucine zipper protein ATHB-6-like
Unigene4844_All	-0.84	-1.32	Nucleoside diphosphate kinase 2
CL288.Contig1_All	-0.78	-1.27	FGGY family of carbohydrate kinase
Unigene596_All	-0.26	-1.12	Calcium-dependent protein kinase 3
Unigene927_All	-0.95	-2.19	Serine/Threonine-protein kinase HT1-like
CL8664.Contig2_All	-0.90	-1.18	Serine/Threonine-protein kinase SAPK10
Unigene16504_All	-0.10	-1.47	Serine/Threonine-protein kinase HT1
Unigene14659_All	-0.57	-1.68	CBL-interacting serine/threonine-protein kinase
CL9890.Contig1_All	-0.80	-1.58	ABC1 family protein
CL8540.Contig1_All	-0.60	-1.55	Cytokinin dehydrogenase 7-like
	Roots		
Unigene625_All	0.57	1.14	Dof zinc finger protein dof1.7-like
CL1607.Contig1_All	0.80	1.68	WRKY 1
Unigene12410_All	0.86	1.67	NAP-like
Unigene2036_All	0.49	1.34	ERF8
Unigene5805_All	0.34	1.33	NAC-like protein 1
Unigene6596_All	1.19	1.46	Heat stress transcription factor B-3
CL4361.Contig1_All	0.34	2.79	MYB39
CL5171.Contig1_All	1.31	2.01	GRAS
Unigene14974_All	1.26	1.74	Transcription factor divaricata-like
CL1857.Contig2_All	0.63	1.48	Cysteine-rich receptor-like protein kinase
Unigene16534_All	0.16	1.29	CDPK substrate protein 1
Unigene917_All	1.02	1.52	Calcium-binding protein
CL3924.Contig3_All	2.34	3.40	Calcium ion binding protein
CL1302.Contig2_All	0.82	1.40	Calmodulin
Unigene11733_All	-0.60	-1.43	Bzip transcription factor ATB2
Unigene23089_All	-1.57	-1.92	Histidine kinase 1
CL1082.Contig2_All	-0.63	-1.36	Protein phosphatase 2C
Unigene22250_All	-0.94	-1.91	Auxin response factor
Unigene6666_All	-0.93	-1.72	Auxin-responsive protein IAA27-like isoform 1
CL10244.Contig1_All	-0.82	-1.58	Brassinosteroid insensitive-associated receptor kinase 1 precursor
Unigene2780_All	-2.09	-2.54	Gibberellin-regulated protein 4-like

Table S6 The continuously up-regulated or down-regulated unigenes related to ROS-scavenging in shoots and roots in *H. ammodendron* under sorbitol treatment

Gene ID	Log2 ratio		Functional annotation
	6h/0h	24h/0h	
Shoots			
Unigene8527_All	3.33	4.29	Thioredoxin H
Unigene23370_All	2.59	3.35	Thioredoxin H-type 3
CL10307.Contig1_All	0.32	1.92	Thioredoxin H9-like
Unigene11984_All	1.67	2.20	Peroxidase
Unigene3727_All	3.33	3.72	APX
CL1674.Contig1_All	0.50	1.43	Peroxidase 5
Unigene20893_All	0.55	1.40	Peroxidase 4 Precursor
Unigene20145_All	0.26	2.36	Glutathione S-transferase
CL2806.Contig1_All	0.34	1.38	Monodehydroascorbate reductase
Unigene23361_All	0.42	2.34	Mitochondrial AOX1a
CL7345.Contig1_All	1.47	2.59	Chloroplastic polyphenol oxidase precursor
CL9446.Contig2_All	-0.68	-1.53	Peroxidase prx12 precursor
Roots			
Unigene23361_All	0.17	1.63	Mitochondrial AOX1a
CL483.Contig1_All	0.44	1.33	Thioredoxin H-type
CL2269.Contig1_All	6.15	7.29	TRX
CL5540.Contig2_All	1.00	3.81	Glutathione-s-transferase δ
CL2340.Contig1_All	0.66	1.64	GSTU45
Unigene3727_All	-0.41	-1.10	APX

Table S7 The continuously up-regulated or down-regulated unigenes related to general metabolism in shoots and roots in *H. ammodendron* under sorbitol treatment

Function	Gene ID	Log2 ratio		Functional annotation
		6h/0h	24h/0h	
	Shoots			
Photosynthesis	CL495.Contig2_All	6.34	7.83	Plastid-targeted protein 4
	CL8116.Contig2_All	0.36	1.07	Photosystem II protein CP43
	Unigene22249_All	1.56	1.66	Photoperiod responsive protein
	CL582.Contig2_All	0.26	1.17	Ferrochelatase
	Unigene22307_All	0.54	1.05	Fructose-bisphosphate aldolase, cytoplasmic isozyme
	CL8287.Contig2_All	0.34	1.37	Fructose-bisphosphate aldolase
	CL10309.Contig2_All	2.18	2.76	UDP-glucose 4-epimerase
	Unigene15376_All	0.28	1.07	Formate dehydrogenase
	Unigene7727_All	0.35	1.03	6-Phosphogluconate dehydrogenase decarboxylating
	CL3800.Contig1_All	0.26	1.61	Neutral invertase-like protein
	Unigene29308_All	-0.53	-1.94	Ribulose-1,5-bisphosphate carboxylase oxygenase
	CL1478.Contig2_All	-0.73	-1.20	Ribulose-bisphosphate carboxylase
	Unigene19857_All	-0.48	-1.39	Phosphoenolpyruvate carboxylase kinase
	Unigene20826_All	-0.22	-6.98	Chloroplast oxygen-evolving enhancer protein 1

	Unigene25759_All	-1.06	-1.38	Chloroplast outer envelope membrane protein
	Unigene2293_All	-0.21	-1.66	Chlorophyll a/b-binding protein
	Unigene23997_All	0.13	-1.51	Chloroplast RNA binding protein
	Unigene22119_All	-0.62	-1.23	PsaH (chloroplast)
	Unigene23921_All	-0.64	-1.39	NADP-dependent malic enzyme
	CL7791.Contig2_All	-0.47	-1.49	NADP-dependent malic enzyme
	CL9028.Contig1_All	-0.11	-1.21	Plastid ribosomal protein PRPL5
	Unigene34045_All	-1.31	-2.29	Ferredoxin-1, chloroplastic
	CL2761.Contig2_All	-0.67	-1.58	Transketolase, chloroplastic
	Unigene51492_All	2.05	-6.17	Transaldolase
	CL1236.Contig1_All	-0.59	-1.30	Sedoheptulose-1,7-bisphosphatase, chloroplastic
	CL8781.Contig2_All	-0.23	-1.39	Pyruvate orthophosphate dikinase
Cell wall	Shoots			
	CL4502.Contig1_All	5.17	8.17	Glucan endo-1,3-beta-glucosidase
	Unigene5573_All	1.94	1.94	Glucan endo-1,3-beta-D-glucosidase
	Unigene13421_All	0.96	2.96	Xyloglucan endotransglycosylase/hydrolase 1
	CL602.Contig1_All	0.15	1.93	Xyloglucan endotransglucosylase
	CL602.Contig2_All	0.12	1.52	Xyloglucan endotransglucosylase
	Unigene14716_All	4.75	7.29	Laccase-12-like
	Unigene25164_All	2.22	2.22	Inositol oxygenase 2-like
	CL5568.Contig5_All	1.08	1.96	Expansin-like A1-like
	Unigene17630_All	0.63	1.09	Expansin-like B1
	Unigene20699_All	2.00	2.02	Polygalacturonase inhibitor protein
	Unigene13337_All	5.75	7.58	Polygalacturonase inhibitor protein
	Unigene9154_All	0.95	1.46	Cinnamate 4-hydroxylase
	Unigene15310_All	0.42	3.50	Cinnamate 4-hydroxylase
	CL5562.Contig1_All	1.19	1.31	Caffeic acid 3-O-methyltransferase
	CL9978.Contig1_All	1.00	2.66	Callose synthase 3
	Unigene4771_All	1.04	3.06	Lipid binding protein
	CL2344.Contig1_All	0.36	1.64	Lipid transfer protein
	CL2344.Contig2_All	0.90	1.65	Lipid transfer protein
	Unigene15391_All	2.52	3.70	Lipid/Phospholipid transfer protein(PLTP)
	Unigene6345_All	0.96	1.83	Lipid-transfer protein type 2
	Unigene1022_All	0.81	2.00	Lipase class 3-like protein
	Unigene5087_All	-1.01	-1.48	Pectinesterase inhibitor
	Roots			
	Unigene4771_All	0.24	1.00	Lipid binding protein
	Unigene15310_All	1.06	2.44	Cinnamate 4-hydroxylase

	Unigene14669_All	-0.41	-1.10	Cinnamyl alcohol dehydrogenase
	CL7785.Contig2_All	-1.24	-2.90	Polyphenoloxidase
	Shoots			
Secondary metabolism	Unigene9650_All	1.39	2.65	Leucoanthocyanidin dioxygenase-like
	Unigene23979_All	4.17	7.03	Chalcone synthase
	CL8370.Contig2_All	2.84	3.39	Polyneuridine-aldehyde esterase
	Unigene18566_All	0.13	1.60	Nicotinamidase
	Unigene3151_All	1.02	1.03	Homogentisate phytyltransferase
	CL6009.Contig1_All	1.95	2.36	Cytochrome P450
	Unigene5962_All	2.38	2.99	Cytochrome P450 94A1-like
	Unigene25199_All	5.75	7.12	Cytochrome P450
	Unigene17304_All	2.07	2.18	Cytochrome P450 83B1-like
	Unigene34432_All	0.71	2.27	Cytochrome P450
	Unigene34834_All	1.24	1.72	Cytochrome c oxidase subunit 2
	Unigene5961_All	1.53	1.98	Cytochrome P450
	Unigene18122_All	-0.24	-1.21	Farnesyl diphosphate synthase
	CL970.Contig1_All	-0.24	-1.43	Geranyl pyrophosphate synthase
	CL7562.Contig2_All	-1.06	-1.05	Solanesyl diphosphate synthase
	Unigene20429_All	-0.59	-1.01	Isoflavone reductase homolog
	Roots			
	Unigene5962_All	0.25	3.20	Cytochrome P450 94A1-like
	Unigene40893_All	0.45	1.23	2-Nitropropane dioxygenase family
	Unigene6592_All	-1.14	-4.07	Geranylgeranyl diphosphate reductase

Table S8 The continuously up-regulated or down-regulated unigenes related to stress response genes in shoots and roots in*H. ammodendron* under sorbitol treatment

Gene ID	Log2 ratio		Functional annotation
	6h/0h	24/0h	
Shoots			
Unigene7846_All	0.00	2.02	Universal stress protein YxiE-like
Unigene10745_All	2.12	2.59	Defensin-like protein
Unigene18131_All	0.54	1.92	Multidrug and toxin extrusion protein 2-like
CL10126.Contig3_All	2.69	4.03	Germin-like protein
CL9957.Contig2_All	0.29	2.13	Resistance protein
CL301.Contig1_All	0.69	1.25	L-allo-threonine aldolase-like
Unigene3656_All	1.23	2.40	Phosphoethanolamine N-methyltransferase
Unigene23534_All	0.64	2.85	Phosphoethanolamine N-methyltransferase
Unigene5998_All	0.34	1.87	ACD-sHSPs-like protein
CL2793.Contig4_All	2.12	3.74	thaumatin-like protein
CL2793.Contig2_All	1.74	3.41	thaumatin-like protein
Unigene4757_All	2.47	3.24	Putative dehydrin
Unigene25984_All	0.37	1.15	Delta-1-pyrroline-5-carboxylate synthase
Unigene25313_All	1.56	3.50	LEA protein D-29
Unigene3754_All	2.46	5.01	LEA protein group 8 protein
CL8223.Contig2_All	5.17	7.44	LEA
CL1418.Contig1_All	0.61	2.35	alpha-1,4-glucan-protein synthase

CL7583.Contig2_All	1.38	2.80	Nbs-lrr resistance protein
Unigene16430_All	1.20	3.34	Pathogenesis-related protein
Unigene28987_All	0.00	7.44	Nbs-lrr resistance protein
Unigene23106_All	-0.11	2.53	Nbs-lrr resistance protein
Unigene10905_All	0.32	1.45	Reticulon-like protein B8 isoform 3
Unigene5096_All	0.25	1.13	BED finger-nbs-lrr resistance protein
CL9422.Contig1_All	1.25	1.67	Pathogenesis-related protein R major form -like protein E22
CL841.Contig1_All	1.52	2.19	Pathogenesis-related protein
CL5692.Contig1_All	7.17	7.64	Pathogenesis-related protein 10a
Unigene3614_All	7.08	6.13	Nbs-lrr resistance protein
Unigene25274_All	1.61	1.59	Disease resistance response protein 206-like
Unigene6693_All	1.00	2.07	Chitinase, class V
CL6655.Contig1_All	1.07	1.37	Chitinase
Unigene7676_All	-0.56	-1.02	Proline-rich protein
Unigene25254_All	-0.79	-1.24	20 kDa chaperonin, chloroplastic
Unigene20539_All	-0.59	-1.21	Chaperone protein DNAJ 16-like
Unigene12360_All	-0.87	-1.76	Thaumatin-like protein-like
CL7515.Contig1_All	-0.39	-1.57	Major latex like protein homolog
Unigene6717_All	-0.15	-1.28	Bifunctional polymyxin resistance protein ArnA-like
CL2635.Contig3_All	-2.10	-2.79	Momilactone A synthase-like
CL5231.Contig2_All	-0.81	-6.98	Bifunctional dihydroflavonol 4-reductase/flavanone 4-reductase
CL9106.Contig2_All	-0.81	-1.22	Bifunctional dihydroflavonol 4-reductase/flavanone 4-reductase
Roots			
Unigene18131_All	0.69	1.45	Multidrug and toxin extrusion protein 2-like
CL1403.Contig7_All	0.71	1.34	Cold acclimation protein
CL10305.Contig1_All	0.74	2.94	Dehydration responsive protein
Unigene402_All	0.28	1.57	Chaperone protein ClpB3
CL214.Contig1_All	-0.91	-1.15	Anti-microbial protein 2
Unigene17571_All	0.50	1.41	Harpin-induced protein
CL10252.Contig1_All	1.12	1.39	HSPRO
Unigene14394_All	-0.84	-1.62	Choline monooxygenase, chloroplastic-like
CL9677.Contig2_All	-0.50	-2.67	Glutaredoxin-C11
Unigene6283_All	-0.65	-1.27	Actin depolymerizing factor
Unigene25767_All	-2.61	-3.28	Metallothionein type 3
CL9397.Contig1_All	-1.02	-2.75	Salt-induced protein
Unigene26784_All	-0.41	-2.08	Oxalate oxidase-like germin 171
Unigene7697_All	-0.70	-3.20	Salt-induced protein
Unigene6025_All	-0.86	-1.27	NBS-LRR disease resistance protein homologue
CL214.Contig1_All	-0.91	-1.15	Anti-microbial protein 2
